# Limited vaccine-induced CD8^+^ T cell immunity in HIV-infected immunological nonresponders

**DOI:** 10.1172/jci.insight.195458

**Published:** 2025-11-10

**Authors:** Vivien Karl, Anne Graeser, Anastasia Kremser, Liane Bauersfeld, Florian Emmerich, Nadine Herkt, Siegbert Rieg, Susanne Usadel, Bertram Bengsch, Tobias Boettler, Hendrik Luxenburger, Christoph Neumann-Haefelin, Matthias C. Müller, Robert Thimme, Maike Hofmann

**Affiliations:** 1 Department of Medicine II (Gastroenterology, Hepatology, Endocrinology and Infectious Diseases), Freiburg University Medical Center, Faculty of Medicine,; 2 Faculty of Biology, and; 3 Institute for Transfusion Medicine and Gene Therapy, Freiburg University Medical Center, Faculty of Medicine, University of Freiburg, Freiburg, Germany.; 4 Department of Infection Medicine, Medical Service Centre Clotten, Freiburg im Breisgau, Germany.; 5 Signalling Research Centres BIOSS and CIBSS, University of Freiburg, Freiburg, Germany.

**Keywords:** AIDS/HIV, Immunology, Virology, Adaptive immunity, T cells, Vaccines

## Abstract

**BACKGROUND:**

Among people living with HIV (PLWH), immunological nonresponders (INR) fail to adequately restore CD4^+^ T cell counts despite effective antiretroviral therapy (ART), placing them at greater risk for adverse outcomes and reduced vaccine efficacy. We aimed to study the robustness and longevity of vaccine-induced virus-specific cellular immune responses in INR.

**METHODS:**

Virus-specific CD8^+^ T cell responses were analyzed in INR (CD4^+^ T cell count < 300 cells/μL) and immunological responders (IR) (CD4^+^ T cell count > 500 cells/μL), receiving ART, and HIV-uninfected controls following COVID-19 mRNA vaccination and infection. Virus-specific CD8^+^ T cells were characterized using peptide-loaded MHC I tetramer technology, after in vitro expansion and cytokine production assays. Virus-specific CD4^+^ T cells and IgG levels were determined by activation-induced marker (AIM) assay and ELISA, respectively.

**RESULTS:**

We demonstrated that, while long-lasting virus-specific cellular immune responses were generated in INR, CD8^+^ T cell immunity remained limited compared with robust CD4^+^ T cell reactivity. CD8^+^ T cell responses in INR exhibited reduced breadth and frequency, accompanied by altered memory differentiation and suboptimal activation and effector response upon antigen exposure. This deficiency correlated with low CD4^+^ T cell counts, independent of other disease markers, highlighting the pivotal role of CD4^+^ T cells in orchestrating vaccine-induced immunity. Notably, repeated booster vaccinations enhanced virus-specific CD8^+^ T cell responses.

**CONCLUSION:**

INR elicit limited vaccine-induced virus-specific CD8^+^ T cell immunity, but booster vaccinations can enhance these responses, suggesting better immune outcomes with tailored vaccination strategies.

**FUNDING:**

Helmholtz Society, German Research Foundation, Federal Ministry of Education and Research.

## Abstract 2

CD4+ T cell deficiency limits vaccine-induced virus-specific CD8+ T cell immunity in HIV-infected immunological non-responders, which has implications for vaccination strategies in this vulnerable population.

## Introduction

Vaccines are highly effective in reducing the risk of infection and in preventing the development of severe disease. However, while vaccines induce robust immune responses in immunocompetent individuals, immunosuppressed individuals often fail to mount adequate immune responses (1), limiting the effectiveness of vaccines and raising concerns about long-term immunity. Not only memory formation of the humoral response, but also long-lived CD8^+^ T cells are crucial for protective immunity after vaccination. Specifically, long-lived virus-specific CD8^+^ T cells are important antiviral effector cells, as they rapidly mediate viral clearance in the case of an infection and limit disease severity (2–7). For the generation of robust vaccine-induced immunity, coordinated adaptive immune responses are essential (8). Thereby, CD4^+^ T cells play a central role by supporting the development of memory B cells, plasma cells, and antibodies, as well as facilitating CD8^+^ T cell priming and memory formation (9–12).

Given the importance of CD4^+^ T cells in orchestrating strong and lasting vaccine responses, this poses a potential challenge for people living with HIV (PLWH), whose immune system is compromised since CD4^+^ T cells are directly infected by HIV and are eventually reduced in numbers. Antiretroviral therapy (ART) suppresses viral replication and, in most cases, also at least partially restores immune functions. This is typically reflected in rising CD4^+^ T cell counts. However, 15%–30% of treated PLWH fail to recover their CD4^+^ T cells despite viral suppression (13, 14). These individuals are classified as immunological nonresponders (INR) — i.e., they exhibit persistently low CD4^+^ T cell counts. Consequently, they remain at an increased risk of opportunistic infections and other health complications, and they therefore represent a distinctive vulnerable group with particular clinical demand.

Although studies in PLWH have shown that INR can generate immune responses after vaccination, these responses are often reduced, especially with respect to humoral immunity (15–20). While most studies focused on humoral responses, only a limited number investigated cellular immune responses in INR following vaccination (19–21). These studies demonstrated that the overall T cell reactivity is reduced in INR after basic immunization. However, the breadth of CD4^+^ versus CD8^+^ T cells, as well as their strength, phenotype, function, and durability after booster vaccination, are still unclear.

We investigated vaccine-elicited virus-specific cellular immunity in these individuals. To do so, we established a prospective cohort of PLWH including INR and immunological responders (IR) and a control cohort of healthy controls (HC). INR were defined with CD4^+^ T cell counts < 300 cells/μL and IR with CD4^+^ T cell counts > 500 cells/μL. Using COVID-19 mRNA vaccination (BNT162b2/Comirnaty or mRNA-1273/Spikevax) and SARS-CoV-2 infection as model systems, we analyzed the robustness and longevity of vaccine-induced virus-specific CD4^+^ and CD8^+^ T cell immunity in INR versus IR. Overall, our findings suggest that INR are able to form durable virus-specific cellular immune responses. However, the virus-specific CD8^+^ T cell immunity in INR was limited and linked to their reduced CD4^+^ T cell counts with implications for vaccination/boost strategies in this vulnerable patient group.

## Results

### Robust virus-specific CD4^+^ T cell responses in INR.

**Figure 1:**
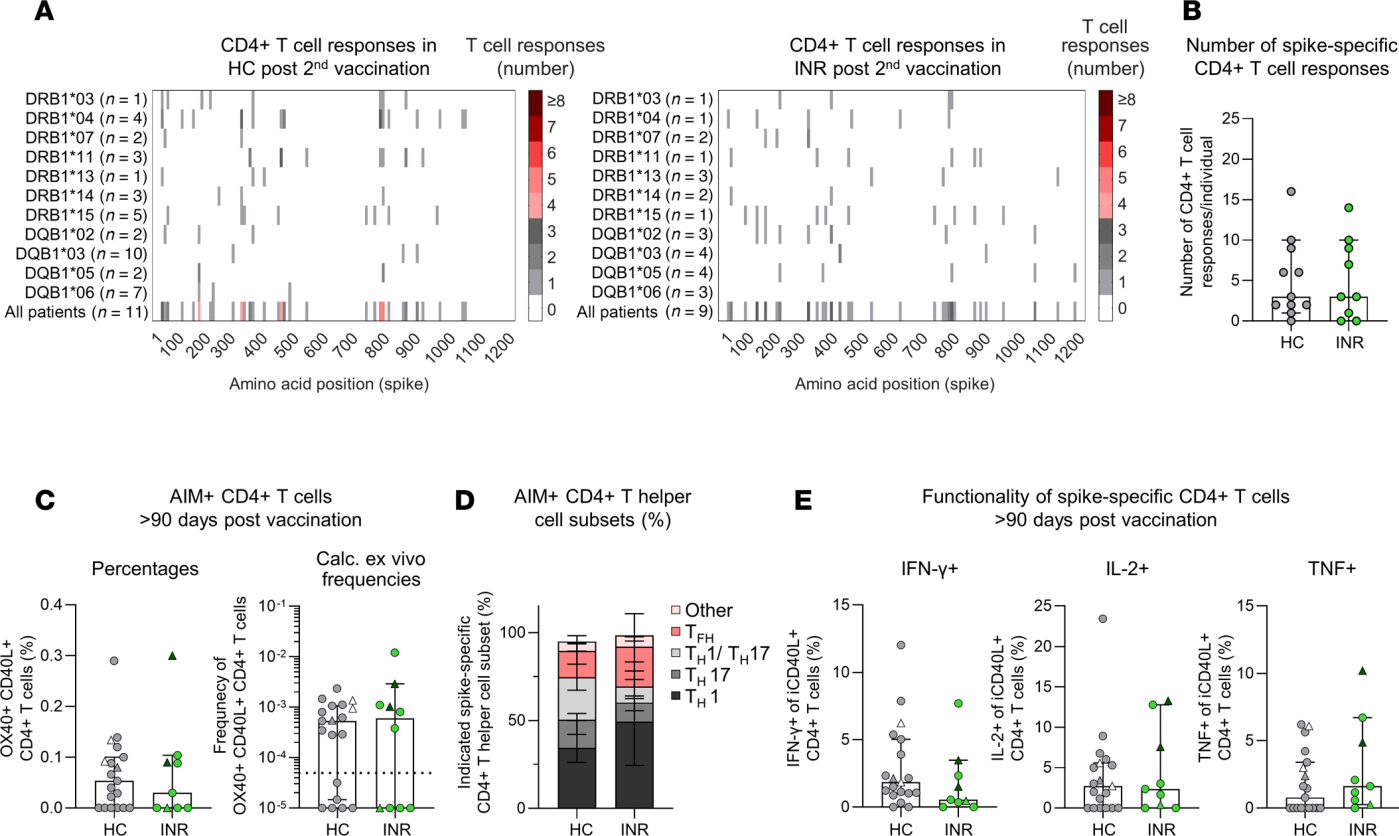
Comparable spike-specific CD4^+^ T cell responses in immunological nonresponders (INR) and healthy controls (HC). (**A**) Number of spike-specific CD4^+^ T cell responses to overlapping peptides (OLPs) of the spike protein detected in HC (*n* = 11) and INR (*n* = 9) > 20 days post second vaccination. Number of tested individuals (per HLA allotype and in total) and location of these epitopes within the spike protein are indicated. (**B**) Number of CD4^+^ T cell responses per individual induced by stimulation with OLPs. (**C**) Percentages (left) and calculated ex vivo frequencies (right) of OX40^+^CD40L^+^ (AIM^+^) nonnative CD4^+^ T cells nonnaive CD4^+^ T cells in HC (*n* = 19) and INR (*n* = 9) > 90 days after second (gray; green) or third (white; dark green) mRNA vaccination. Percentages and frequencies are shown after subtracting the signal detected in paired unstimulated samples. (**D**) Percentages of nonnaive AIM^+^ CD4^+^ Th cell subsets are shown in HC (*n* = 12) and INR (*n* = 4). Tfh: CXCR5^+^; Th1: CXCR3^+^CCR6^–^; Th1/ Th17: CXCR3^+^CCR6^+^; Th17: CXCR3^–^CCR6^+^; Other: CXCR3^–^CCR6^–^. (**E**) Intracellular cytokine production of spike-reactive nonnaive CD4^+^ T cells, defined by intracellular CD40L (iCD40L) expression. Percentages show the indicated intracellular cytokine production by iCD40L^+^CD4^+^ T cells after stimulation with a pool of spike-derived OLPs. Values are shown after subtracting the signal detected in paired unstimulated samples. Gray and green indicate time points after second vaccination. White and dark green indicate time points after third vaccination. Median values are depicted with 95% CI error bars. Statistical analysis was performed with a 2-tailed Mann-Whitney *U* test (**B**, **C**, and **E**). Circles indicate vaccine-induced CD4^+^ T cell responses. Triangles indicate hybrid immunity.

To examine whether INR still mount antigen-specific CD4^+^ T cell immunity or not, we analyzed the breadth of the spike-specific CD4^+^ T cell repertoire after COVID-19 mRNA vaccination. For this purpose, we collected time point–matched samples obtained from INR, IR, and HC (Supplemental Figure 1, A and B, and Supplemental Tables 1–5; supplemental material available online with this article; https://doi.org/10.1172/jci.insight.195458DS1) and stimulated PBMCs with a pool of overlapping peptides (OLPs) covering the entire SARS-CoV-2 WT spike-protein, and expanded the cells for 14 days. After in vitro expansion, we restimulated the cells with pools containing 4 OLPs each, followed by analysis for intracellular IFN-γ production. Subsequently, to determine those spike-derived peptides of the pool that elicited an IFN-γ response in CD4^+^ T cells, we restimulated the cells with the single OLP of positive pools. Once we had identified the positive OLP, we investigated whether there were any predescribed optimal epitopes located within the T cell–triggering OLPs that were restricted by the HLA class II alleles expressed by the respective individual. If no matching optimal epitopes were previously described, we performed an in silico analysis to predict the most likely epitope. Using this comprehensive approach, we identified similar breadth and number of spike-specific CD4^+^ T cell responses in INR and HC (Figure 1, A and B). Of note, IR also showed a similar spike-specific CD4^+^ T cell repertoire and number of spike-specific CD4^+^ T cell responses after COVID-19 mRNA vaccination compared with HC (Supplemental Figure 1, C–E). Since CD4^+^ T cells are not a homogeneous group but differentiate into distinct subsets with specialized roles, we next examined CD4^+^ T cell polarization (Supplemental Figure 2A). In particular, we analyzed CXCR5^+^ T follicular helper (Tfh) cells in peripheral blood as well as CXCR5^–^ Th1 (CCR6^–^CXCR3^+^), Th1-like Th17 (CCR6^+^CXCR3^+^), Th17 (CCR6^+^CXCR3^–^) and CCR6^–^CXCR3^–^ cells. Thereby, we detected reduced frequencies of Th1-like Th17 cells among the overall CD4^+^ T cell population in both INR and IR (Supplemental Figure 2B). To study the CD4^+^ T cell subsets on an antigen-specific level, we stimulated the PBMCs with the spike-derived OLP pool for 24 hours. Virus-specific CD4^+^ T cells were identified by dual expression of the activation-induced markers (AIM), OX40, and CD40L, and after subtracting the signal of unstimulated control samples (Supplemental Figure 2A). Since we detected similar AIM^+^CD4^+^ T cell responses following 2 vaccinations and after ≥ 3 antigen contacts in HC, IR, and INR, we decided to pool these time points for subsequent analyses (Supplemental Figure 3A). INR, IR, and HC showed comparable AIM^+^CD4^+^ T cell responses (Figure 1C and Supplemental Figure 3B). Furthermore, spike-reactive AIM^+^CD4^+^ T cells were predominantly of the Th1 phenotype, which plays a key role in antiviral response (Figure 1D and Supplemental Figure 3C). Analyses of the intracellular cytokine production (IFN-γ, IL-2, and TNF) of the virus-specific CD4^+^ T cells revealed comparable functional capacities in INR, IR, and HC (Figure 1E and Supplemental Figure 3, D and E). Taken together, despite the differences in the overall Th subset distribution, INR mount robust and functionally competent virus-specific memory CD4^+^ T cell immunity.

### Restricted breadth of virus-specific CD8^+^ T cell responses in INR and IR.

**Figure 2:**
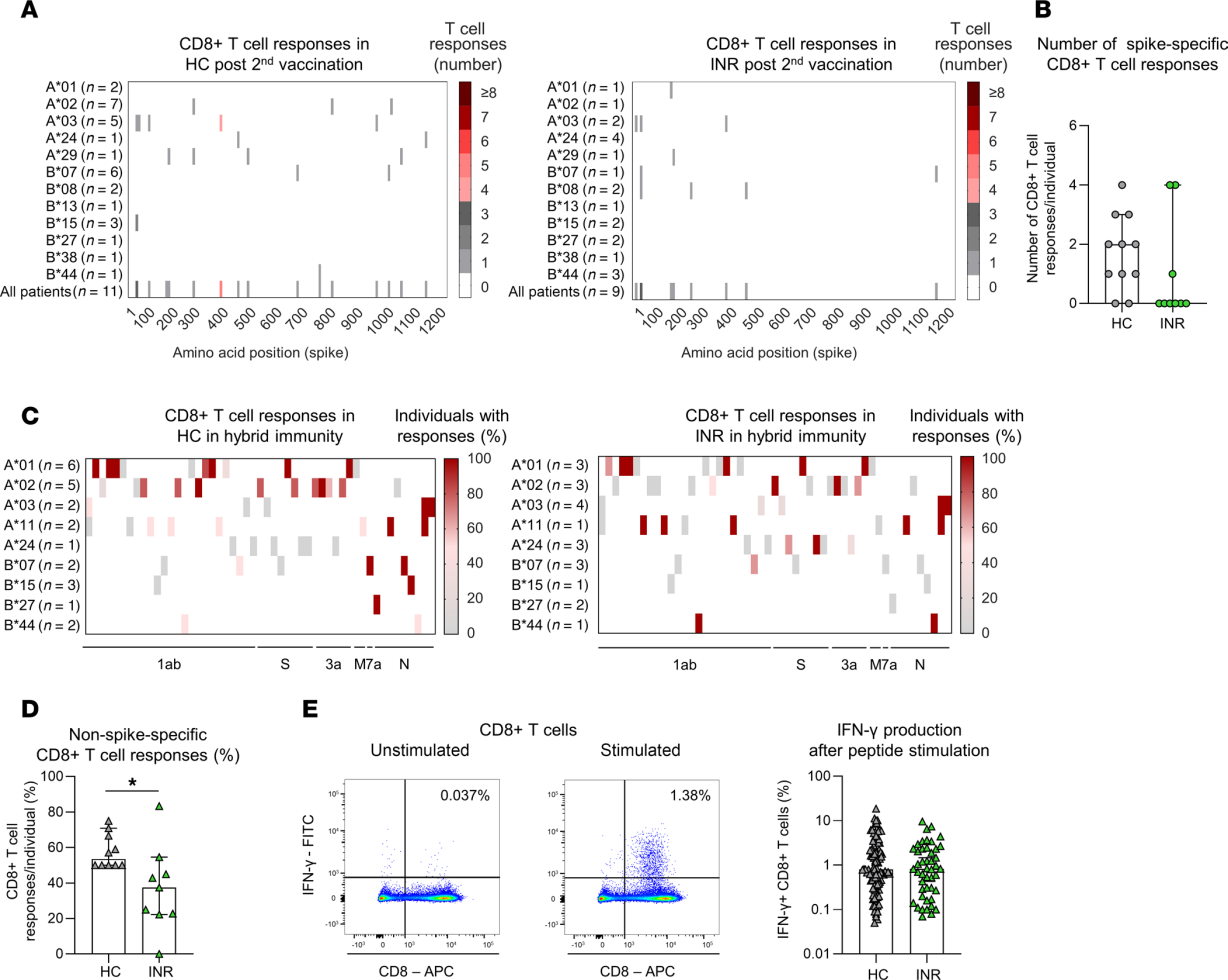
Restricted virus-specific CD8^+^ T cell repertoire after vaccination and infection in INR compared with HC. (**A** and **B**) Spike-specific CD8^+^ T cell responses of HC (*n* = 11) and INR (*n* = 9) > 20 days after second vaccination. (**A**) Number of spike-specific CD8^+^ T cell responses to OLPs of the spike protein. Number of tested individuals (per HLA allotype and in total) and location of these epitopes within the spike protein are indicated. (**B**) Number of spike-specific CD8^+^ T cell responses per individual induced by stimulation with OLPs. (**C** and **D**) HLA-matched SARS-CoV-2–specific CD8^+^ T cell responses of HC (*n* = 10) and INR (*n* = 9) after vaccination and infection (hybrid immunity). (**C**) Percentages of SARS-CoV-2–specific CD8^+^ T cell responses against epitopes within the complete WT SARS-CoV-2 proteome. These CD8^+^ T cell epitopes have been described to be restricted by the indicated HLA allotypes. (**D**) Percentages of non-spike–specific CD8^+^ T cell responses per individual induced by stimulation with predescribed, optimal CD8^+^ T cell epitopes. (**E**) Representative dot plots of unstimulated and stimulated CD8^+^ T cells. Bar graph displays intracellular IFN-γ production after peptide-specific stimulation. Values are shown after subtracting the signal detected in unstimulated samples. Median values are depicted with 95% CI error bars. Statistical analysis was performed with a 2-tailed Mann-Whitney *U* test (**B**, **D**, and **E**). Circles indicate vaccine-induced CD8^+^ T cell responses. Triangles indicate hybrid immunity.*P < 0.05.

Next, we addressed the question, whether the effector arm is altered in INR after vaccination. For this, we first analyzed spike-specific IgG titers after 2 vaccinations as well as after ≥ 3 antigen contacts in INR, IR, and HC. We found a tendency toward lower levels of spike-specific IgG in INR after 2 vaccinations but a clear increase after additional vaccination or infection, reaching levels similar to IR and HC (Supplemental Figure 4A). In addition to the humoral effector response, we also investigated the breadth of the spike-specific CD8^+^ T cell repertoire. For this purpose, we again stimulated PBMCs obtained from INR, IR, and HC, with the pool of spike-spanning OLPs. After 14 days of in vitro expansion, we restimulated the cells with pooled OLPs (4 OLPs in each pool) and subsequently identified the single OLP that elicited an IFN-γ response. IFN-γ–producing, virus-specific CD8^+^ T cells were detectable in INR. However, the spike-specific CD8^+^ T cell repertoire showed a restricted breadth compared with HC (Figure 2A). This was evident in INR as well as in IR (Figure 2A and Supplemental Figure 4, B and C). Accordingly, a reduced number of responses per individual were detected in INR and IR (Figure 2B and Supplemental Figure 4D). To additionally investigate CD8^+^ T cell responses targeting epitopes from non-spike viral proteins, we used PBMCs of INR, IR, and HC, who were also infected with SARS-CoV-2 earlier and, therefore, underwent hybrid immunization, and we stimulated these cells with a pool of 54 peptides, which have previously been described to be immunodominant SARS-CoV-2–specific CD8^+^ T cell epitopes. Both INR and IR exhibited SARS-CoV-2–specific CD8^+^ T cells that targeted different viral epitopes including non-spike–derived epitopes. However, the non-spike–specific CD8^+^ T cell response also showed a restricted breadth in INR and IR compared with HC (Figure 2C and Supplemental Figure 5A). In line with this, a reduced number of non-spike–specific CD8^+^ T cell responses per individual were again found in INR and IR (Figure 2D and Supplemental Figure 5B). Nevertheless, if INR and IR mounted virus-specific CD8^+^ T cell responses, their SARS-CoV-2–specific CD8^+^ T cells produced similar intracellular IFN-γ levels as the ones from HC, suggesting comparable cytokine production (Figure 2E and Supplemental Figure 5C). Taken together, these results indicate that both INR and IR elicit reduced anti-viral CD8^+^ T cell immunity with restricted breadth.

### Reduced frequencies and altered subset diversification of virus-specific memory CD8^+^ T cells in INR.

**Figure 3:**
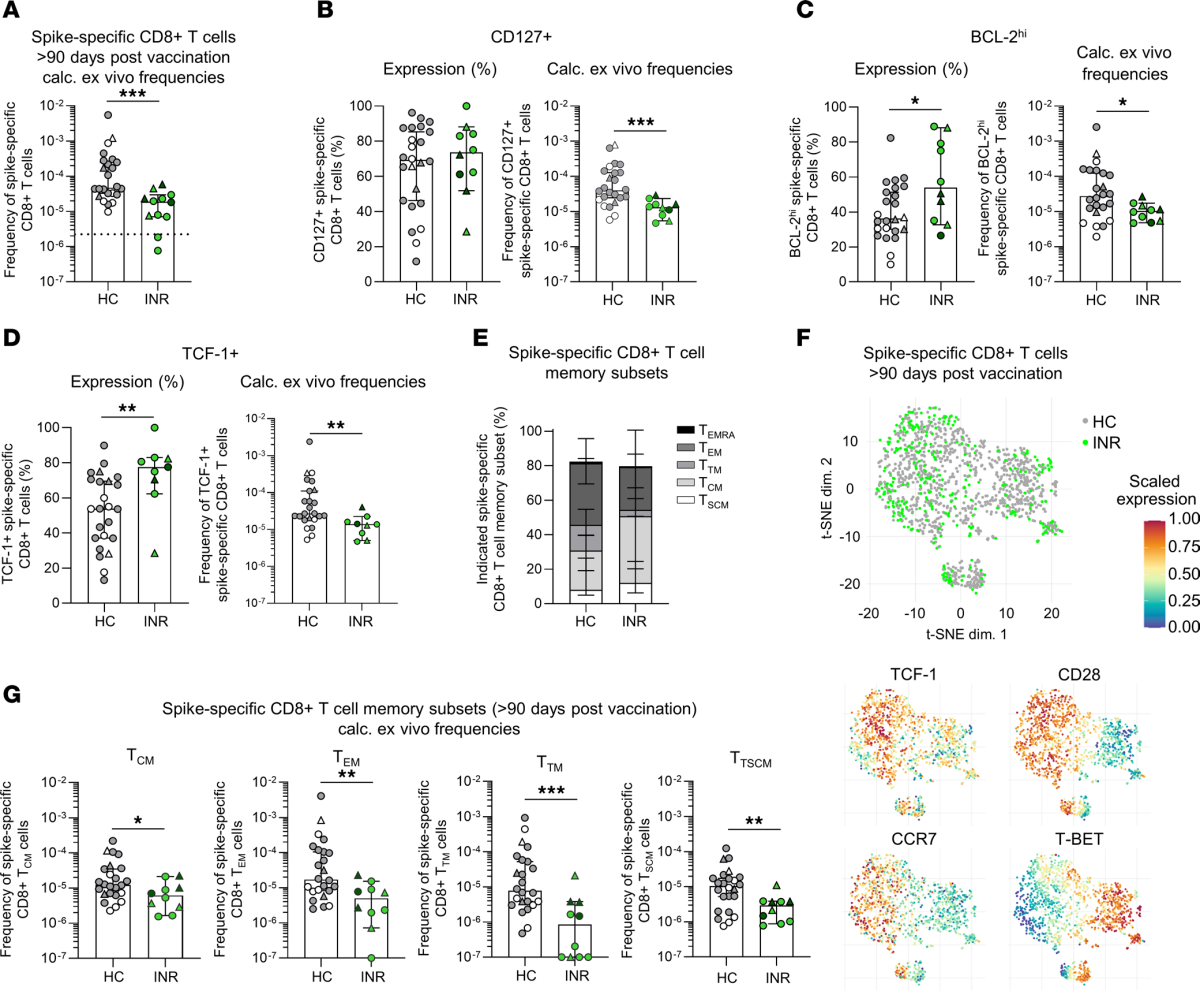
Reduced spike-specific CD8^+^ T cell memory formation in INR compared with HC. (**A**) Calculated ex vivo frequencies of spike-specific CD8^+^ T cells in HC (*n* = 24) and INR (*n* = 12) > 90 days after second (gray; green) or third (white; dark green) mRNA vaccination. (**B**–**D**) Percentages and calculated ex vivo frequencies of CD127^+^ (HC *n* = 24; INR *n* = 10) (**B**), BCL-2^hi^ (HC *n* = 24; INR *n* = 10) (**C**), and TCF-1^+^ (HC *n* = 24; INR *n* = 9) (**D**) nonnaive spike-specific CD8^+^ T cells. (**E**) Percentages of spike-specific CD8^+^ T cell memory subsets in HC (*n* = 24) and INR (*n* = 10). T_EMRA_, terminally differentiated effector memory cells reexpressing CD45RA; T_EM_, effector memory; T_TM_, transitional memory; T_CM_, central memory; T_SCM_, stem cell–like memory. (**F**) t-SNE representation of flow cytometry data depicting spike-specific CD8^+^ T cells > 90 days after second and third mRNA vaccination. Gray, HC after first boost (*n* = 17) and after second boost (*n* = 6); green, INR post first boost (*n* = 7) and after second boost (*n* = 2). (**G**) Calculated ex vivo frequencies of spike-specific CD8^+^ T cell memory subsets in HC (*n* = 24) and INR (*n* = 10). Gray and green indicate time points post second vaccination. White and dark green indicate time points post third vaccination. Median values are depicted with 95% CI error bars. Statistical analysis was performed with a 2-tailed Mann-Whitney *U* test (**A**–**D** and **G**). Circles indicate vaccine-induced CD8^+^ T cell responses. Triangles indicate hybrid immunity. *P < 0.05, **P < 0.01, ***P < 0.001.

To examine whether the formation of the virus-specific memory CD8^+^ T cell response is also altered in INR on a single epitope level, we studied CD8^+^ T cells targeting A*01/S_865_, A*02/S_269,_ or A*03/S_378_ epitopes of the SARS-CoV-2 spike protein > 90 days after vaccination. To increase the detection sensitivity of the spike-specific CD8^+^ T cells, we performed peptide-loaded MHC-class I tetramer–based enrichment (Supplemental Figure 6A). We detected similar frequencies of spike-specific CD8^+^ T cells following 2 vaccinations and after ≥ 3 antigen contacts in HC, IR, and INR (Supplemental Figure 6B). Therefore, we decided to combine these time points for subsequent analyses. Comparison of spike-specific CD8^+^ T cell frequencies among the cohorts revealed significantly lower frequencies in INR compared with HC and IR (Figure 3A and Supplemental Figure 6C). Reduced frequencies were also observed in INR compared with HC when the virus-specific CD8^+^ T cell responses were divided into vaccine-induced and hybrid immunization responses (Supplemental Figure 6D). In contrast, IR elicited comparable frequencies to HC (Supplemental Figure 7A). To investigate the overall emergence of virus-specific memory CD8^+^ T cells, we next analyzed the expression of the IL7R alpha chain (CD127), the antiapoptotic molecule BCL-2, and the transcription factor TCF-1, all of which are expressed by memory CD8^+^ T cells. Robust expression of all 3 markers was detectable in spike-specific CD8^+^ T cells, indicating that INR are also capable of forming memory CD8^+^ T cells (Figure 3, B–D, and Supplemental Figure 7, B–D). However, INR, but not IR, exhibited significantly lower frequencies of CD127^+^, BCL-2^hi^, and TCF-1^+^ spike-specific CD8^+^ T cells compared with HC (Figure 3, B–D, and Supplemental Figure 7, B–D). Furthermore, the subset diversification—and, with this, the quality of spike-specific memory CD8^+^ T cells—was different in INR to HC. More precisely, early differentiated T cell subsets such as central memory (T_CM_), were increased, but effector (T_EM_) and transitional memory (T_TM_) CD8^+^ T cells were decreased in INR (Figure 3E and Supplemental Figure 8A). This shift toward early differentiated memory CD8^+^ T cells in INR was also detectable when analyzing the marker expression in an unbiased way, including expression of TCF-1, CD28, CCR7, CD45RA, T-BET, CD95, CD127, CD27, BCL-2, and CD38. T-distributed stochastic neighbor embedding (t-SNE) analyses confirmed on a more comprehensive phenotypic level that spike-specific CD8^+^ T cells obtained from INR were less differentiated as highlighted by the expression of CD28 and TCF-1, whereas the spike-specific CD8^+^ T cells of HC showed an increased expression of T-BET, thus reflecting a more differentiated effector cell state (Figure 3F and Supplemental Figure 8B). In contrast to this, IR and HC displayed similar phenotypic characteristics of spike-specific memory CD8^+^ T cells, indicated by complete intermingling in t-SNE analyses (Supplemental Figure 8, C and D). Although INR were able to form virus-specific memory CD8^+^ T cells, they still showed significantly lower frequencies of each memory subset compared with HC (Figure 3G). This was not the case for IR (Supplemental Figure 8E). Hence, our data collectively indicate that INR are capable of forming robust virus-specific memory CD8^+^ T cell responses, albeit at lower frequencies and with different subset distribution.

### Robust recall capacities of virus-specific CD8^+^ T cells in INR.

**Figure 4:**
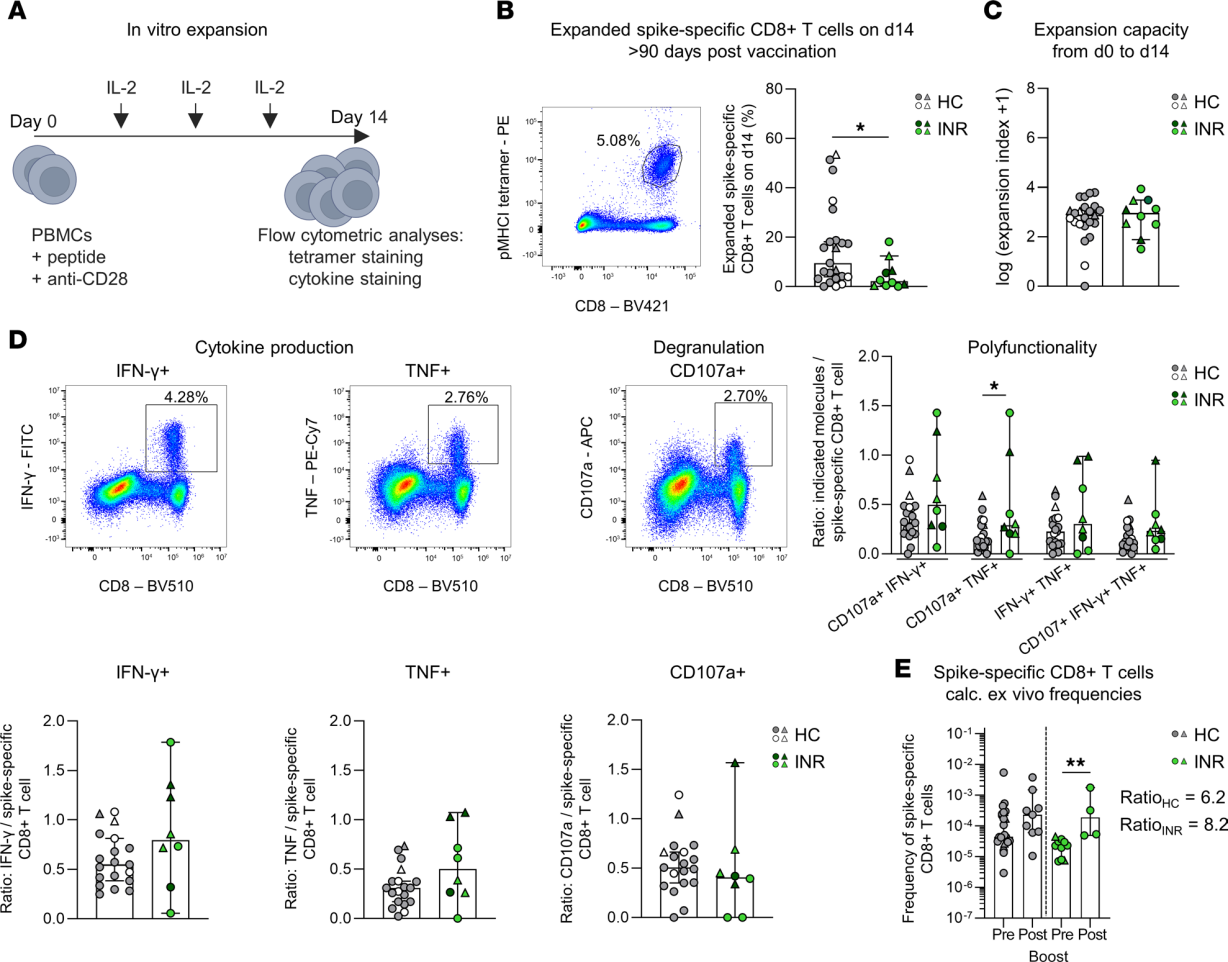
Similar in vitro and in vivo recall responses of spike-specific CD8^+^ T cells in INR and HC. (**A**) Experimental setup of in vitro expansion of peptide-specific CD8^+^ T cells. (**B**) Representative dot plot and percentages of expanded spike-specific CD8^+^ T cells on day 14. Results are shown for HC (*n* = 23) and INR (*n* = 10) > 90 days after second (gray; green) or third (white; dark green) mRNA vaccination. (**C**) In vitro expansion capacity of spike-specific CD8^+^ T cells from day 0 to day 14 in HC (*n* = 23) and INR (*n* = 10) > 90 days after second (gray; green) or third (white; dark green) mRNA vaccination. (**D**) Representative dot plots and percentages of cytokine-producing CD8^+^ T cells, including polyfunctional populations, are shown relative to the frequency of spike-specific CD8^+^ T cells after in vitro expansion > 90 days after second (gray; green) or third (white; dark green) mRNA vaccination. (**E**) Calculated ex vivo frequencies of spike-specific CD8^+^ T cells before (HC *n* = 23; INR *n* = 10) and 7–14 days after (HC *n* = 9; INR *n* = 4) third vaccination. The ratio is calculated of the median frequency before versus after third vaccination of HC and INR, respectively. Median values are depicted with 95% CI error bars. Statistical analysis was performed with a 2-tailed Mann-Whitney *U* test (**B**–**D**), a 2-way ANOVA with Šídák’s multiple-comparison test to compare the polyfunctionality (**D**), and a Kruskal-Wallis test and Dunn’s multiple-comparison test to compare the frequencies of spike-specific CD8^+^ T cells pre versus post booster vaccination (**E**). Circles indicate vaccine-induced CD8^+^ T cell responses. Triangles indicate hybrid immunity. *P < 0.05, **P < 0.01.

To assess whether virus-specific memory CD8^+^ T cell immunity is also functionally impaired in INR, we analyzed the in vitro recall capacities of the virus-specific memory CD8^+^ T cells obtained from INR, IR and HC > 90 days after vaccination. After 14 days of expansion, INR, but not IR, exhibited significantly lower frequencies of spike-specific CD8^+^ T cells compared with HC (Figure 4, A and B, and Supplemental Figure 9A). However, when comparing the expansion capacity of the spike-specific CD8^+^ T cells from day 0 to day 14, comparable levels of expansion were observed in INR, IR, and HC (Figure 4C and Supplemental Figure 9B). In addition, to measure the effector function per cell, we evaluated the spike-specific intracellular production of IFN-γ, TNF, and the degranulation marker CD107a, as well as their coproduction, in relation to the frequency of spike-specific CD8^+^ T cells after expansion. Spike-specific CD8^+^ T cells of INR, similar to those of IR, produced comparable amounts of IFN-γ, TNF, and CD107a, including coproduction, to HC, indicating robust in vitro functionality (Figure 4D and Supplemental Figure 9, C and D). Next, we assessed the recall responses in vivo. Analyses of the spike-specific CD8^+^ T cells shortly before and 6–15 days after a third vaccination showed a boost in frequencies, suggesting that virus-specific CD8^+^ T cells of INR and IR exhibited in vivo comparable recall responses compared with HC (Figure 4E and Supplemental Figure 10). Overall, these data show that INR exhibit robust recall responses.

### Stable long-term virus-specific CD8^+^ T cell immunity in INR.

**Figure 5:**
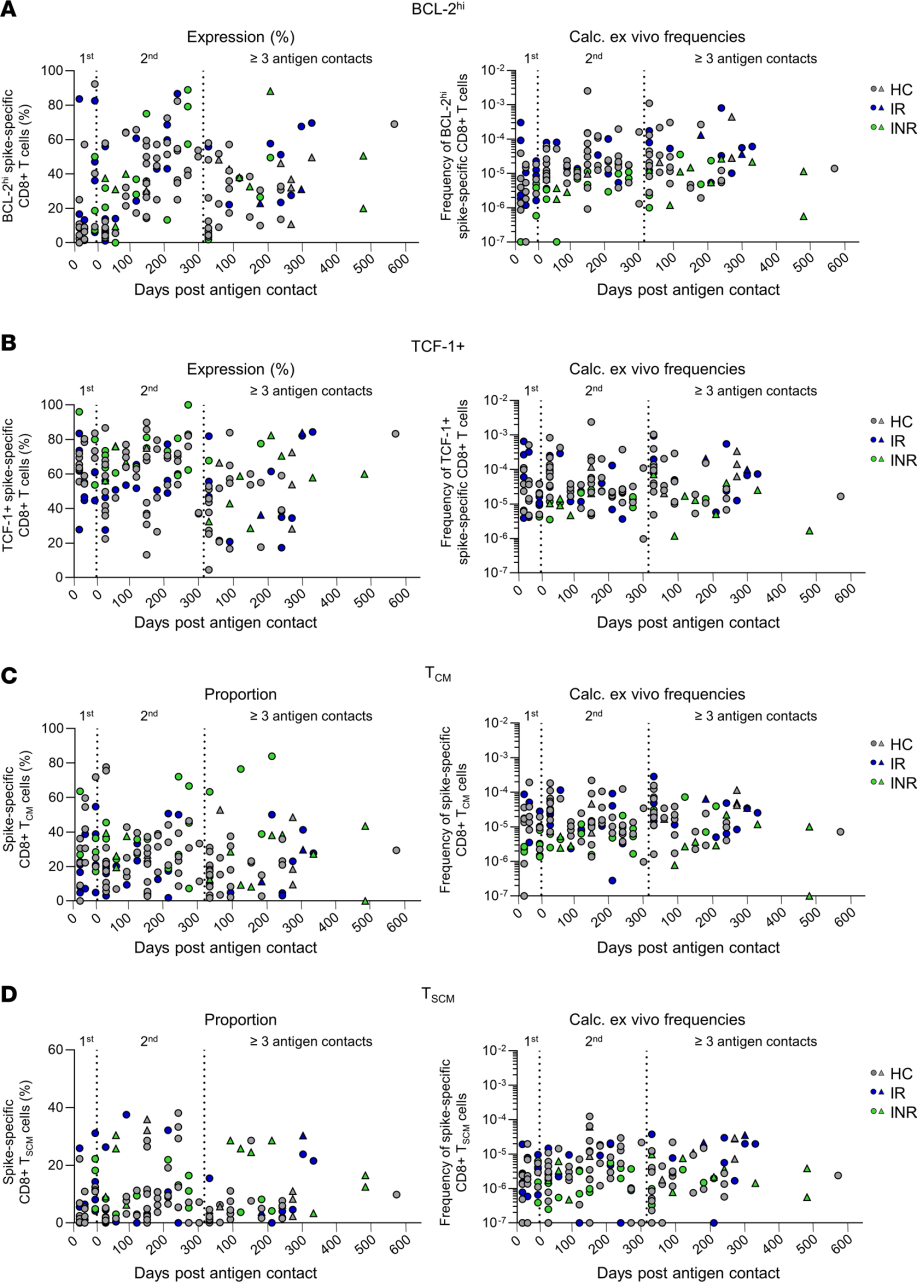
Stable long-term memory of spike-specific CD8^+^ T cells in INR, IR, and HC. (**A**–**D**) Percentages and calculated ex vivo frequencies of BCL-2^hi^ (**A**), TCF-1^+^ (**B**), T_CM_ (**C**), and T_SCM_ (**D**) spike-specific CD8^+^ T cells in the course of one to more than 3 antigen contacts in HC (*n* = 24), IR (*n* = 6), and INR (*n* = 10). Circles indicate vaccine-induced CD8^+^ T cell responses. Triangles indicate hybrid immunity.

Next we investigated the dynamics and longevity of the virus-specific CD8^+^ T cell immunity in INR. We longitudinally analyzed spike-specific CD8^+^ T cells highly expressing the memory markers BCL-2 and TCF-1 over the course of 1 to ≥ 3 antigen contacts in INR, IR, and HC. Spike-specific BCL-2^hi^ and TCF-1^+^CD8^+^ T cells were already induced after the first antigen contact (Figure 5, A and B). Each antigen contact resulted in a transient reduction in the proportion of virus-specific CD8^+^ T cells expressing BCL2^hi^ and TCF-1 resembling an effector response. Following this effector response, BCL2^hi^ and TCF-1^+^ virus-specific CD8^+^ T cells leveled back to the proportions present before the respective antigen contact indicating stable effector/memory dynamics also in INR. In line with this, stable frequencies—a surrogate for cell counts—of BCL-2^hi^ and TCF-1^+^ spike-specific CD8^+^ T cells were detectable for more than 300 days after respective antigen contacts (Figure 5, A and B). Additionally, we studied the emergence and dynamics of spike-specific CD8^+^ T_CM_ and stem cell–like memory T (T_SCM_) cells, which are both essential for long-term immunity. Proportions of spike-specific CD8^+^ T_CM_ and T_SCM_ cells increased until a resting memory phase was reached (Figure 5, C and D). Frequencies of spike-specific CD8^+^ T_CM_ and T_SCM_ cells were again stable beginning from the second antigen contact for more than 300 days after each of multiple antigen contacts in INR, IR, and HC (Figure 5, C and D). In summary, these results suggest that INR are able to form durable and stable virus-specific CD8^+^ T cell memory.

### Virus-specific CD8^+^ T cell immunity in INR and IR is linked to their CD4^+^ T cell counts.

**Figure 6:**
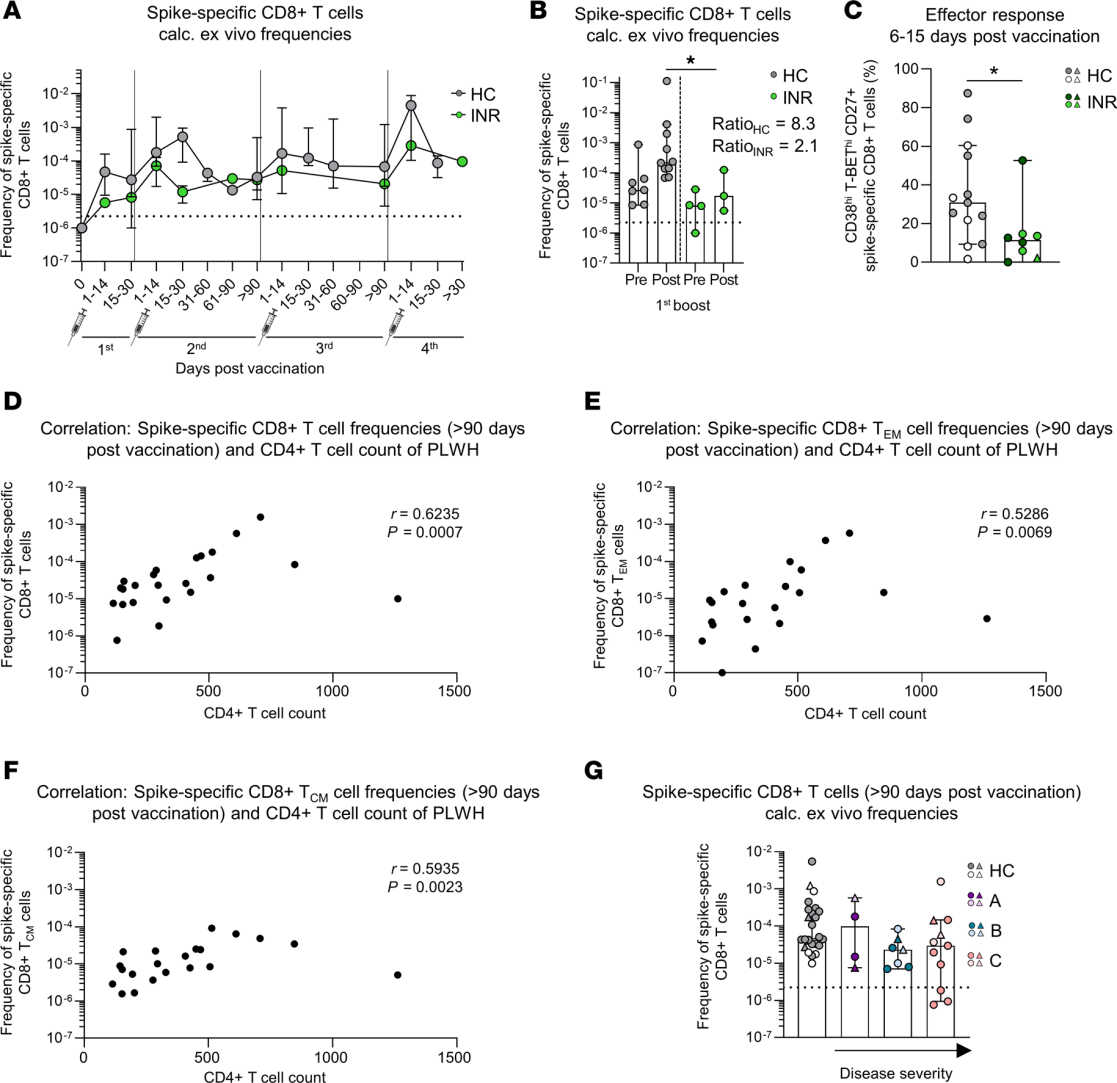
Spike-specific CD8^+^ T cell responses are linked to CD4^+^ T cell counts. (**A**) Calculated ex vivo frequencies of spike-specific CD8^+^ T cells throughout first, second, third, and fourth mRNA vaccination. Median is depicted of HC (*n* = 9 after third vaccination, of which *n* = 3 are after fourth vaccination) and INR (*n* = 4 after third vaccination, of which *n* = 1 after fourth vaccination). (**B**) Calculated ex vivo frequencies of spike-specific CD8^+^ T cells before (HC, *n* = 7; INR, *n* = 4) and 6–15 days after (HC, *n* = 10; INR, *n* = 3) first boost. The ratio is calculated of the median frequency before versus after boost of HC and INR, respectively. (**C**) CD38^hi^T-BET^hi^CD27^+^ expression within spike-specific nonnaive CD8^+^ T cells 6–15 days after booster vaccination in HC (*n* = 13) and INR (*n* = 8). Gray and green indicate time points after second vaccination. White and dark green indicate time points after third vaccination. (**D**–**F**) Correlation of CD4^+^ T cell counts and frequencies of spike-specific CD8^+^ T cells (**D**), CD8^+^ T_EM_ (**E**), and CD8^+^ T_CM_ cells (**F**) in PLWH. *n* = 23 (**D**); *n* = 21 (**E** and **F**). (**G**) Calculated ex vivo frequencies of spike-specific CD8^+^ T cells in HC (*n* = 24) and PLWH (*n* = 20) > 90 days after second (dark colors) or third (light colors) mRNA vaccination. Letters indicate disease severity. A, asymptomatic; B, symptomatic, not AIDS-defining; C, AIDS-defining illness. Median values are depicted with 95% CI error bars (**A**–**C** and **G**). Statistical analysis was performed with a Kruskal-Wallis test and Dunn’s multiple-comparison test (**B** and **G**), a 2-tailed Mann-Whitney *U* test (**C**), and Spearman correlation (**D**–**F**). Circles indicate vaccine-induced CD8^+^ T cell responses. Triangles indicate hybrid immunity. *P < 0.05.

Although the stability and, thus, longevity of the virus-specific CD8^+^ T cell response does not appear to be impaired in the INR, we still observed significantly lower frequencies at later time points after vaccination (Figure 3A). This led us to question whether the CD8^+^ T cell response might have already been compromised during the initial activation phase. To address this question, we longitudinally analyzed the overall frequencies of spike-specific CD8^+^ T cells in INR, IR and HC (Supplemental Figure 1A). Similar dynamics of induction, expansion, and contraction of the spike-specific CD8^+^ T cell response were found (Figure 6A and Supplemental Figure 11A). However, in INR, the frequencies of spike-specific CD8^+^ T cells appeared to be reduced from the beginning, and a lower amplitude was observed after boost vaccination, indicating an activation/effector response defect (Figure 6A). To examine the activation and effector response more in detail, we compared the frequencies of spike-specific CD8^+^ T cells before and 6–15 days after first boost. INR, but not IR, showed significantly lower frequencies early after vaccination compared with HC (Figure 6B and Supplemental Figure 11B). In addition, a smaller boost effect was observed on the spike-specific CD8^+^ T cell frequencies of INR compared with HC (Figure 6B). In accordance with these results, during the peak response, INR showed significantly lower expression of the activation and effector markers CD38^hi^, T-BET^hi^, and CD27 on spike-specific CD8^+^ T cells (Figure 6C), while IR demonstrated similar expression levels compared with HC (Supplemental Figure 11C). Since we found reduced virus-specific CD8^+^ T cell responses only in INR, who all had CD4^+^ T cell counts < 300 cells/μL, we were wondering whether there is a link between the virus-specific CD8^+^ T cell response and the exact CD4^+^ T cell counts. To address this point, we analyzed the frequencies of the spike-specific CD8^+^ T cells of PLWH in relation to their respective CD4^+^ T cell counts and did, in fact, find a positive correlation, as it was also evident for the subset analyses of spike-specific CD8^+^ T_EM_ and T_CM_ cells (Figure 6, D–F). To further verify this association, we divided the cohort of PLWH into 2 groups, based on their CD4/CD8 ratio. This ratio is an additional biomarker of disease progression and an indicator of treatment response (22). HIV infection typically inverts this ratio, resulting in a CD4/CD8 ratio < 1, whereas a normal ratio is > 1 (22). In line with our previous findings, PLWH with a ratio < 1 had reduced frequencies of spike-specific CD8^+^ T cells, whereas the frequencies of PLWH with a ratio > 1 were similar to those of HC (Supplemental Figure 11D). Notably, these significant differences in the frequencies of virus-specific CD8^+^ T cells were apparent only when the cohort of PLWH was divided based on their CD4^+^ T cell counts (Figure 3A and Supplemental Figure 6C, and Supplemental Figure 11D) but not when grouped according to their symptom classification (Figure 6G). This classification is based on the CDC guidelines, where category C represents the most severe cases and category A refers to asymptomatic individuals. In summary, virus-specific CD8^+^ T cell responses are reduced in INR, likely due to impaired activation/effector response that appears to be closely linked to the CD4^+^ T cell counts.

## Discussion

Here, we used COVID-19 mRNA vaccination as a model to explore whether long-lasting CD4^+^ and CD8^+^ T cell immunity can be generated and modulated through vaccination in INR with severe immune dysfunction. Our findings provide insights into the nature of vaccine-induced cellular immunity within this highly vulnerable patient group with implications for clinical management.

First, we showed that a functionally competent virus-specific CD4^+^ and CD8^+^ T cell memory is formed in INR following vaccination. However, compared with HC, INR exhibited a reduced pool of virus-specific memory CD8^+^ T cells — an effect not observed in IR. Thus, the cellular effector arm appears to be less efficient in INR, adding to the previously reported less durable humoral response in PLWH (23). In contrast, IR established a robust virus-specific CD4^+^ and CD8^+^ T cell immunity after vaccination, almost similar to HC. These latter observations are in line with previous studies on SARS-CoV-2–specific T cell immunity in IR (23, 24) showing a robust T cell response after vaccination. These findings clearly highlight that the vaccine-induced cellular immune response differs in PLWH with reduced efficacy in INR compared with IR, fostering the vulnerability of INR.

Second, the reduced frequency of vaccine-elicited virus-specific memory CD8^+^ T cells in INR was clearly linked to the low CD4^+^ T cell count and not to other disease parameters. CD4^+^ T cell help for CD8^+^ T cell responses ranges from effects on priming, proliferation, and memory formation (25). Thus, a possible reason for the reduced generation of memory CD8^+^ T cells could be insufficient activation during the initial priming, which can impair clonal expansion and result in lower frequencies of both effector and memory CD8^+^ T cells (26–30). In fact, we observed that INR exhibited lower frequencies of virus-specific CD8^+^ T cells already after first vaccination, supporting impaired initial activation. Moreover, we also detected an attenuated effector virus-specific CD8^+^ T cell response after booster vaccination, pointing toward a broader activation defect beyond initial priming. CD8^+^ T cell activation requires 3 distinct signals: (a) antigen recognition by the T cell receptor, (b) binding of costimulatory molecules, and (c) cytokines, which are produced by activated DCs (31). Studies in mouse models have shown that CD4^+^ T cells are involved in these 3 signaling pathways. For example, it has been reported that CD4^+^ T cells can “license” DCs to interact with and activate CD8^+^ T cells by inducing increased antigen presentation and upregulation of costimulatory molecules, highlighting the central role of CD4^+^ T cells in CD8^+^ T cell activation in general (25, 32–34). In addition, by producing cytokines such as IL-2, CD4^+^ T cells facilitate CD8^+^ T cell proliferation, differentiation, and survival (35–37). In line with this, we found that less differentiated memory subsets were present within the pool of virus-specific memory CD8^+^ T cells in INR, all of which had CD4^+^ T cell counts < 300 cells/μL, suggesting also in humans a crucial role for CD4^+^ T cells in the differentiation and maintenance of virus-specific CD8^+^ T cell responses. Thus, our study demonstrates, in a clinical setting, that sufficient CD4^+^ T cell help is probably an essential factor for the robust induction and proper differentiation of antigen-specific CD8^+^ T cells in response to mRNA vaccination.

However, CD4^+^ T cell count is probably not the only factor affecting the vaccine-elicited virus-specific CD8^+^ T cell response, as in addition to INR, we also show that IR with normalized CD4^+^ T cell counts exhibited a reduced breadth of virus-specific CD8^+^ T cell responses. There are several possible determinants beyond CD4^+^ T cell help affecting CD8^+^ T cell immunity in PLWH. For example, HIV infection also diminishes the pool of naive CD8^+^ T cells (38), thus possibly limiting new CD8^+^ T cell responses to other pathogens. Furthermore, although ART effectively suppresses viral replication, viral antigen may persist at certain sites (e.g., lymphatic tissues) (39), leading to chronic immune activation and inflammation, which can also contribute to CD8^+^ T cell dysfunction. In addition, in response to the chronic immune activation, immunosuppressive molecules may be produced (40), resulting in overall reduced antiviral immune responses. Hence, even when the CD4^+^ T cell counts are restored by ART, there are still several other mechanisms of immune dysregulation associated with HIV that may partially impair vaccine-induced CD8^+^ T cell immunity.

Third, despite reduced CD4^+^ T cell counts and the persistent immunodeficiency induced by the HIV infection, we were still able to detect long-living virus-specific memory CD8^+^ T cells in INR and IR. Persistence of vaccine-induced CD8^+^ T cell responses has been shown in PLWH for up to 6 months (23, 41). Of note, these studies were performed on PLWH on stable ART with CD4 counts > 350 cells/μL. Thus, our data expand on these findings, demonstrating that also INR with CD4^+^ T cell counts < 300 cells/μL elicit durable CD8^+^ T cell immunity after mRNA vaccination. Moreover, we observed that the dynamics of vaccine-induced CD8^+^ T cell responses in INR and IR were similar to those of HC—more specifically, each vaccination induced a transient boost in the frequencies of virus-specific CD8^+^ T cells.

Lastly, in contrast to HC (5), we detected a small but significant overall increase in virus-specific CD8^+^ T cell frequencies in INR after the third antigen contact. Similar observations have been reported analyzing the overall T cell reactivity, not discriminating between CD4^+^ and CD8^+^ T cells, in INR (21). In line with other reports (42), we also observed an overall boost in spike-specific antibody titers in INR after more than 3 antigen contacts. These observations show that repetitive antigen stimulation — e.g., by booster vaccination — can enhance robustness of the adaptive immune response especially in INR. Future studies are needed to determine whether the durable, vaccine-induced virus-specific CD8^+^ T cell responses — capable of being repeatedly boosted — are also observed in PLWH without treatment-mediated viral suppression; whether there are sex-specific differences; and whether they reflect immune dynamics within lymphoid tissues. Nevertheless, our findings underscore the benefit of frequent booster vaccinations for INR, a particularly vulnerable group within PLWH. To maximize protection during periods of heightened exposure risk, these boosters should be strategically timed with seasonal patterns. In sum, our results carry important implications for tailored vaccination strategies in PLWH stratified according to their extent of immune dysfunction.

## Methods

### Sex as a biological variable.

Our study included samples from males and females. However, sex was not considered in the analysis.

### Cohort.

Initially, 70 PLWH were recruited in an outpatient care setting in Freiburg (Germany) and provided written informed consent. In total, 44 of these patients were included in different experiments, based on the availability of samples, specific HLA types, infection state, and defined time points following vaccination. In total, the overall cohort consisted of 17 INR (14 males, 3 females; median age: 48 years; age range: 28-73 years) and 27 IR (23 males, 4 females; median age: 49; age range: 28-89 years). In addition, 39 HIV-uninfected HC (17 males, 22 females; median age: 35 years; age range: 21-78 years) were recruited at the Freiburg University Medical Center, Germany. For direct comparisons in respective analyses we only included age-matched donors. Blood, plasma, and serum samples were collected during the course of 3 to 4 mRNA vaccinations (BNT162b2/Comirnaty or mRNA-1273/Spikevax) and after hybrid immunization, which refers to the combination of vaccination and natural infection. SARS-CoV-2 infections were detected by positive PCR-testing from oropharyngeal swab or confirmed by seropositivity for anti-SARS-CoV-2 nucleocapsid IgG. Detailed information on the participants are provided in Supplemental Tables 1–5. HLA genotypes were determined by next-generation sequencing.

### PBMC isolation.

Blood samples were collected in EDTA blood collection tubes. PBMCs were isolated by density centrifugation with lymphocyte separation medium (Pancoll separation medium, PAN Biotech GmbH), and stored at –80°C until further processing. To use PBMCs for analyses, cells were thawed in prewarmed RPMI cell culture medium supplemented with 10% fetal calf serum, 1% penicillin/streptomycin, 1.5% 1 M HEPES (all purchased from Thermo Fisher Scientific), and 50 U/mL Benzonase (Sigma).

### Peptides and tetramers.

Optimal peptides were produced with an unmodified N-terminus and an amidated C-terminus with standard Fmoc chemistry and exhibited > 70 % purity (Genaxxon Bioscience) (Supplemental Table 6). In addition, a total of 182 OLPs covering the entire SARS-CoV-2 spike sequence (GenBank accession no. MN908947.3) were generated as 18-mer overlapping by 7 amino acids. Of note, OLPs were designed based on the amino acid sequence modification (K986P, V987P) to “stabilize” spike in the approved mRNA vaccine. To generate tetramers, SARS-CoV-2 spike peptides (A*01/S_865_ LTDEMIAQY, A*02/S_269_ YLQPRTFLL and A*03/S_378_ KCYGVSPTK) were loaded on HLA class I easYmers (immunAware) according to the manufacturer’s instructions. Subsequently, peptide-loaded monomers were tetramerized by conjugation to phycoerythrin-coupled (PE, Agilent) or allophycocyanin-coupled (APC, BD Bioscience) streptavidin according to the manufacturer’s instructions.

### AIM assay and intracellular cytokine staining (ICS).

For each condition of the AIM assay and ICS, 1x10^6^ PBMCs were used, resulting in a total of 6x10^6^ PBMCs per donor. First, for each condition, 20 % of the PBMCs were seeded into 96-well U-bottom plates. Subsequently, cells were stimulated for 1 h at 37°C with costimulation (anti-CD28, BD Biosciences) and either a pool of 182 OLPs (10 μg/mL) covering the entire spike protein of SARS-CoV-2, phytohemagglutinin M (PHA-M, 10 μg/mL) as a positive control, or with an equimolar amount of DMSO as a negative control. In addition, cells used for the AIM assay were treated with CD40 blocking antibody (0.5 μg/mL, Miltenyi Biotec) to enable surface staining of CD40L. All conditions were additionally stained with antibodies targeting CCR7, CCR6, CXCR3 and CXCR5. After 1 h of incubation, cells were washed with RPMI cell culture medium and the remaining, unstimulated 80 % of the PBMCs were added (total cell number per well about 1x10^6^ cells). Cells were then cultured for 24 h at 37°C. Cells for AIM assay were cultured in the presence of costimulation, antibodies targeting chemokine receptors, CD40 blocking antibody (0.5 μg/mL, Miltenyi Biotec) and either the spike OLP pool (1 μg/mL), PHA-M (5 μg/mL; positive control) or DMSO (5 mg/mL; negative control). Cells for ICS were cultured in the presence of costimulation, antibodies targeting chemokine receptors, brefeldin A (GolgiPlug, 0.5 μL/mL, BD Biosciences) and either the spike OLP pool (1 μg/mL), PHA-M (5 μg/mL; positive control) or DMSO (5 mg/mL; negative control). After 24 h of incubation, surface and ICS was performed. Frequency of AIM^+^ \CD4^+^ T cells was determined by using the following equation: frequency of AIM^+^CD4^+^ T cells = (number of AIM^+^CD4^+^ T cells)/(number of total live CD4^+^ T cells). For identification of AIM^+^ CD4^+^ T helper cell subsets, only samples with ≥ 10 AIM^+^ nonnaive CD4^+^ T cells were included.

### In vitro expansion of SARS-CoV-2–specific T cells and intracellular IFN-γ staining.

For in vitro expansion with OLPs, 6x10^6^ to 9x10^6^ PBMCs were used and for expansion with optimal epitopes, 3x10^6^ to 6x10^6^ PBMCs were used. First, 20% of the PBMCs were stimulated with a pool of 182 SARS-CoV-2 spike OLP or all 54 optimal epitopes (10 μg/mL) for 1 h at 37°C. Subsequently, cells were washed and remaining, unstimulated PBMCs were added. Cells were then expanded for 12-14 days in complete RPMI culture medium supplemented with recombinant IL-2 (rIL-2, 20 IU/mL, StemCell Technologies). On days 10–12, one part of the cells was stimulated with pooled OLP (50 μM; 45 pools with 4 OLP each). Additionally, cells were treated with an equimolar amount of DMSO as negative control PMA and ionomycin as positive control in the presence of brefeldin A (GolgiPlug, 0.5 μL/mL, BD Biosciences) and IL-2. After 5 h of incubation at 3 °C, surface and intracellular IFN-γ staining was performed. On days 12–14, single OLP of positive pools and HLA-matched optimal CD8^+^ T cell epitopes were tested on the remaining cells in the same manner as described above. Finally, amino acid sequences of positive single OLP were analyzed for predescribed minimal epitopes. If no predescribed minimal epitope was found, the Immune Epitope Database (https://www.iedb.org/) was used to predict the best matching epitope for each HLA type. We used 2 prediction algorithms, ANN 4.0 and NetMHCpan EL 4.123, for 8-mer, 9-mer, and 10-mer peptides with a half maximal inhibitory concentration of < 500 nM). HLA class I binding predictions were made using the IEDB analysis resource ANN aka NetMHC v4.0 tool or the IEDB analysis resource NetMHCpan v.4.0 tool. HLA class II binding predictions were made using the IEDB recommended analysis resource consensus tool 2.22 (smm/nn/sturniolo).

### In vitro expansion of spike-specific CD8^+^ T cells and analyses of effector function.

1.5x10^6^ PBMCs were stimulated with peptides derived from the SARS-CoV-2 spike protein (A*01/S_865_, A*02/S_269_ and A*03/S_378_). All 3 epitopes exhibit low sequence homology with SARS-CoV-1, MERS or common cold coronaviruses (6). Thus, CD8^+^ T cells targeting these epitopes are specific for the spike protein of SARS-CoV-2. In addition, PBMCs were stimulated with anti-CD28 monoclonal antibody (0.5 μg/mL, BD Biosciences) and expanded for 14 days in complete RPMI culture medium supplemented with rIL-2 (20 IU/mL, StemCell Technologies). After 14 days of in vitro expansion, cytokine production and degranulation was induced by stimulation of the cells with spike protein-derived peptides (15 μM) in the presence of anti-CD107a (BD Bioscience) for 1 h at 37°C. Additionally, cells were left untreated as negative control or stimulated with PMA and ionomycin as positive control. After 1 h of incubation, brefeldin A (GolgiPlug, 0.5 μL/mL) and monensin (GolgiStop, 0.5 μL/mL) (both BD Biosciences) were added and incubation was continued for additional 4 h. Afterward, surface and ICS was performed. In addition to the restimulation, expanded cells were stained with peptide-loaded HLA class I tetramers to assess the expansion capacity and functionality of the expanded cells. Expansion index was calculated as described previously (43).

### Magnetic bead-based enrichment of spike-specific CD8^+^ T cells.

SARS-CoV-2 spike-specific CD8^+^ T cells were enriched as described previously (44). In brief, 5x10^6^ to 10x10^6^ PBMCs were stained with APC-conjugated peptide-loaded HLA class I tetramers for 30 min at room temperature. Subsequently, cells were incubated with magnetic anti-APC microbeads for 20 min at 4°C. Magnetically labeled cells were then isolated by positive selection using magnetic-activated cell sorting (MACS) technology (Miltenyi Biotec) according to the manufacturer’s instructions. Enriched spike-specific CD8^+^ T cells were analyzed using multicolor flow cytometry. Frequencies of spike-specific CD8^+^ T cells were calculated as described previously (44). Briefly, first the number of total CD8^+^ T cells within the sample was determined by using the following equation: number of total CD8^+^ T cells = (total number of cells in preenriched sample) x ([live CD8^+^ T cells in the single cell gate in the preenriched sample]/100). Subsequently, frequency of virus-specific CD8^+^ T cells was determined by using the following equation: frequency of virus-specific CD8^+^ T cells = (number of tetramer-positive CD8^+^ T cells in enriched sample)/(number of total CD8^+^ T cells). For phenotypical analyses, only samples with ≥ 5 nonnaive spike-specific CD8^+^ T cells were included. Thus, the average detection limit in this study was 2.24x10^–6^.

### Multiparametric flow cytometry for T cell analyses.

Antibodies that were used for T cell analyses are listed in Supplemental Table 7. Staining of intranuclear and cytoplasmic targets was performed by using FoXP3/Transcription Factor Staining Buffer Set (Thermo Fisher Scientific) and Fixation/Permeabilization Solution Kit (BD Biosciences) according to the manufacturer’s instructions, respectively. After staining, cells were fixed in 2% paraformaldehyde (Sigma) and samples were measured on FACSCanto II or LSRFortessa with FACSDiva software version 10.6.2 (BD Bioscience), or CytoFLEX (Beckman Coulter) with CytExpert Software version 2.3.0.84. Date were further analyzed using FlowJo version 10.7.1 (Treestar).

### Dimensional reduction of multiparametric flow cytometry data.

Flow cytometry data were analyzed with R version 4.2.1 and the Bioconductor CATALYST package (release 3.15). Analyses were performed on gated nonnaive spike-specific CD8^+^ T cells including the markers TCF-1, CD28, CCR7, CD45RA, CD95, CD127, CD27, BCL-2, T-BET, and CD38. Before dimensionality reduction, cell counts were set to a maximum of 50 cells per sample to facilitate visualization. Marker intensities were transformed by arcsinh (inverse hyperbolic sine) with a cofactor of 150. Dimensionality reduction on the transformed data was achieved by t-SNE.

### Determination of IgG levels.

To determine anti-SARS-CoV-2 spike (S) IgG titers, the Euroimmun “Anti-SARS-CoV-2–QuantiVac-ELISA (IgG)” was used according to the manufacturer’s instructions (anti-SARS-CoV-2 S-IgG <35.2 BAU/mL: negative; ≥ 35.2 BAU/mL: positive). To detect anti-SARS-CoV-2 nucleoprotein (N) IgG, the Mikrogen assay “recomWell SARS-CoV-2 (IgG)” was used according to the manufacturer’s instructions (anti-SARS-CoV-2 N-IgG <20 U/mL: negative). Samples were measured on a Tecan’s Spark microplate reader and analyzed with the SparkControl Magellan software version 2.2.

### Statistics.

Statistical analyses were performed using GraphPad Prism software version 10.2.3. Statistical significance was assessed by 2-tailed Mann-Whitney *U* test, Kruskal-Wallis test and Dunn’s multiple-comparison test, Spearman correlation, and 2-way ANOVA with Šídák’s multiple-comparison test. A *P* value less than 0.05 was considered significant.

### Study approval.

Written informed consent was obtained from all study participants. The study was conducted in accordance to federal guidelines, local ethics committee regulations (University of Freiburg, Germany; vote: 322/20, 21-1135 and 383/19; State Medical Association of Baden-Württemberg (Landesärztekammer Baden-Württemberg), Germany; vote: B-F-2017-109) and the Declaration of Helsinki (1975).

### Data availability.

R code to reproduce the analyses of multiparametric flow cytometry data is available at https://github.com/sagar161286/SARSCoV2_specific_CD8_Tcells; commit ID 7f36525. All requests for additional raw and analyzed data and materials will be promptly reviewed by the University of Freiburg Center for Technology Transfer to verify if the request is subject to any intellectual property or confidentiality obligations. Patient-related data not included in the paper were generated as part of clinical examination and may be subject to patient confidentiality. Any data and materials that can be shared will be released via a material transfer agreement. Supporting data are provided with this paper.

## Author contributions

VK designed, performed, and analyzed the experiments. AG, AK, and LB contributed to the experiments. AG and LB helped in analyzing data. FE performed 4-digit HLA-typing by next-generation sequencing. VK, AG, NH, SR, SU, HL, and MCM recruited donors. BB, TB, and CNH contributed to data interpretation. MH designed and supervised the study and contributed to experimental design. VK, RT, and MH interpreted data and wrote the manuscript.

## Funding support

“Virological and immunological determinants of COVID-19 pathogenesis – lessons to get prepared for future pandemics (KA1-Co-02 ‘COVIPA’)”, a grant from the Helmholtz Association’s Initiative and Networking Fund to RT and MH.

- The German Research Foundation (CRC1160 - Project ID: 256073931 - RT, MH, CNH, and BB; TRR179 - Project ID: 272983813 - RT, MH, CNH, BB, HL, and TB; and CRC1479 - Project ID: 441891347 - MH and BB)
- The Federal Ministry of Education and Research, Project ID 01KX2121, NUM 2.0, COVIM to MH and RT
- The IMMediate Advanced Clinician Scientist-Program, Department of Medicine II, Medical Center – University of Freiburg and Faculty of Medicine, University of Freiburg, funded by the Federal Ministry of Education and Research - 01EO2103 (HL)
- The DFG Heisenberg program of the German Research Foundation (project no. HO 5836/2-1) (MH)

## Supplementary Material

Supplemental data

ICMJE disclosure forms

Supporting data values

## Version 1 11/10/2025

Electronic publication
