# Supplementary material for: Limited vaccine-induced CD8+ T cell immunity in HIV-infected immunological nonresponders
Source: JCI Insight. 2025 Nov 10;10(21):e195458. doi: 10.1172/jci.insight.195458 (PMC12643487; doi:10.1172/jci.insight.195458)
Supplement: Supplemental data [file jciinsight-10-195458-s060.pdf]

Supplemental Figure 1

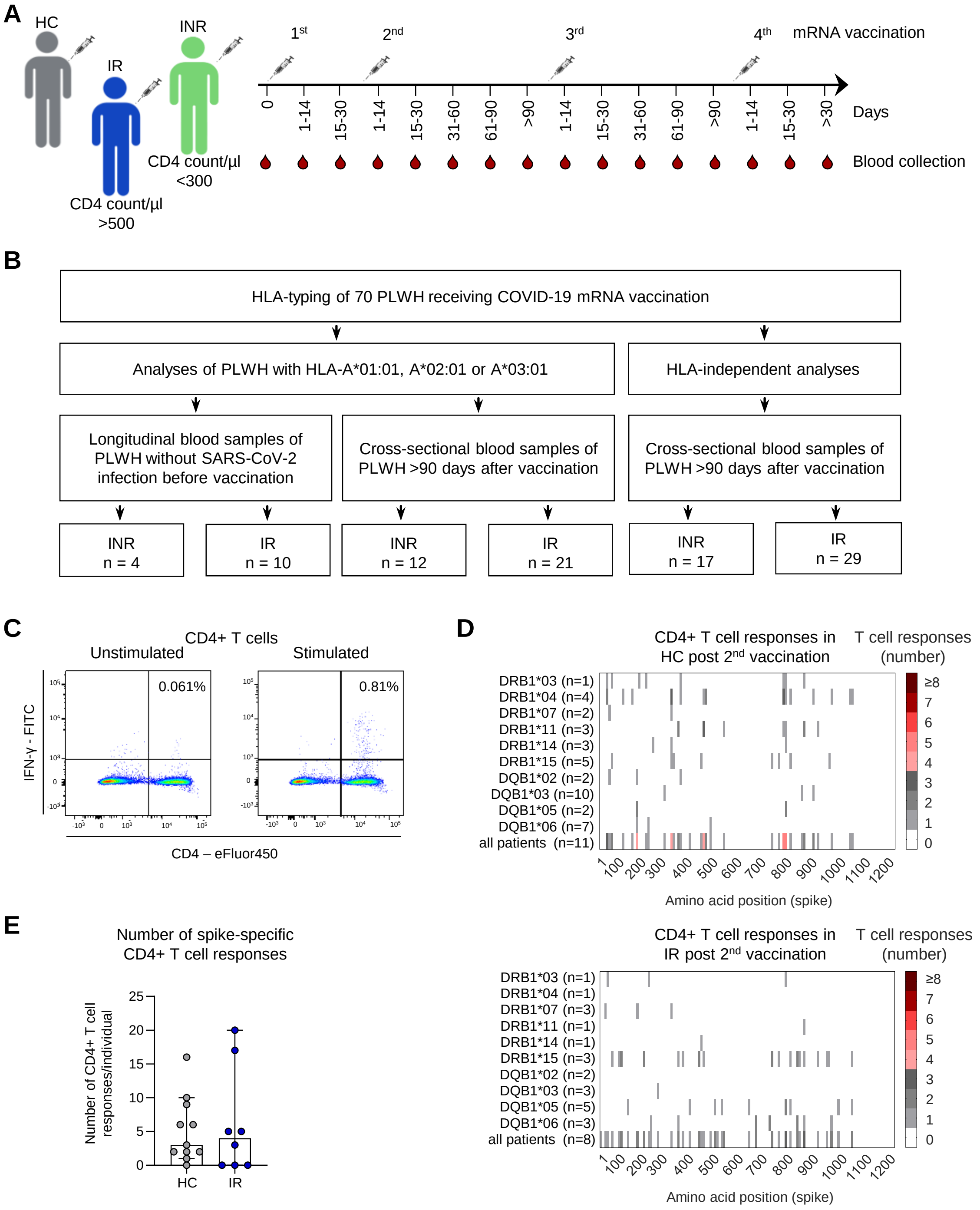

Supplemental Figure 1. Study design and spike-specific CD4+ T cell responses in IR and HC. (**A**) Overview of cohort including vaccination and blood sampling schedule. (**B**) Flow chart displaying the study design and number of individuals included in the analyses. (**C**) Representative dot plots of unstimulated and peptide-stimulated CD4+ T cells. (**D**) Spike-specific CD4+ T cell responses to overlapping peptides (OLPs) of the spike protein detected in HC (n=11) and IR (n=8) >20 days post 2nd vaccination. Number of tested individuals (per HLA allotype and in total) and location of these epitopes within the spike protein are indicated. Data from same HC as in main figure 1A are displayed. (**E**) Number of CD4+ T cell responses per individual induced by stimulation with OLPs. Data from same HC as in main figure 1B are displayed. Median values are depicted with 95% confidence interval error bars. Statistical analysis was performed by a two-tailed Mann–Whitney U test (E).

Supplemental Figure 2

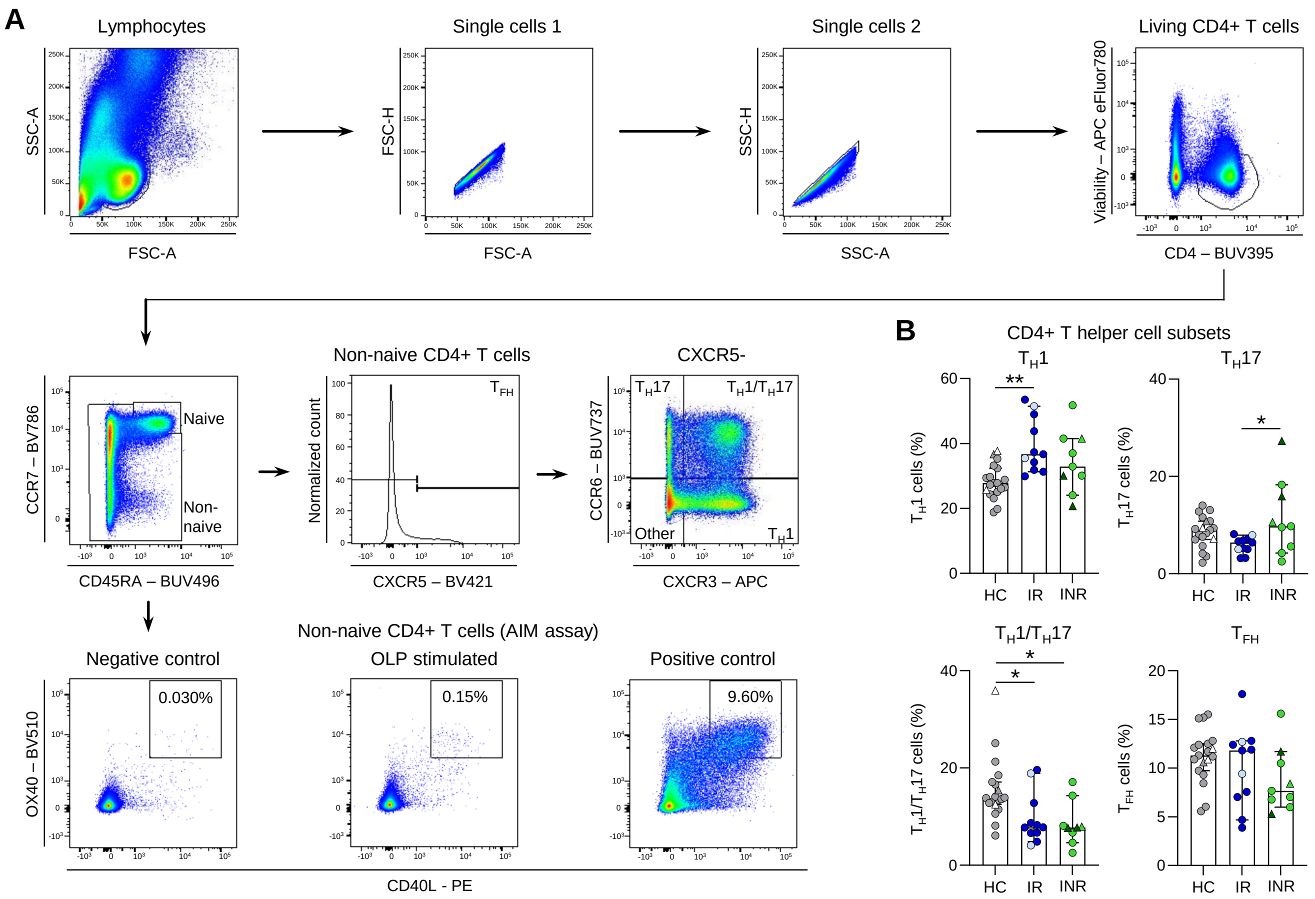

Supplemental Figure 2. Altered CD4+ T cell compartment in INR and IR compared to HC. (**A**) Gating strategy to define activation-induced marker (AIM)+ non-naïve CD4+ T cells and CD4+ T helper (T_H) cell subsets. AIM+ CD4+ T cells were detected by the expression of OX40 and CD40L. (**B**) Non-naïve CD4+ T helper (T_H) cell subsets are shown in DMSO-treated samples of HC (n=19), IR (n=11) and INR (n=9) . T_{FH}: CXCR5+; T_H1: CXCR3+CCR6-; T_H1/ T_H17: CXCR3+CCR6+; T_H17: CXCR3-CCR6+; Other: CXCR3-CCR6-. Grey, blue and green indicate time points post 2nd vaccination. White, light blue and dark green indicate time points post 3rd vaccination. Median values are depicted with 95% confidence interval error bars. Statistical analysis was performed by a Kruskal-Wallis test and Dunn's multiple comparison test (B). Circles indicate vaccine-induced CD4+ T cell responses. Triangles indicate hybrid immunity.

Supplemental Figure 3

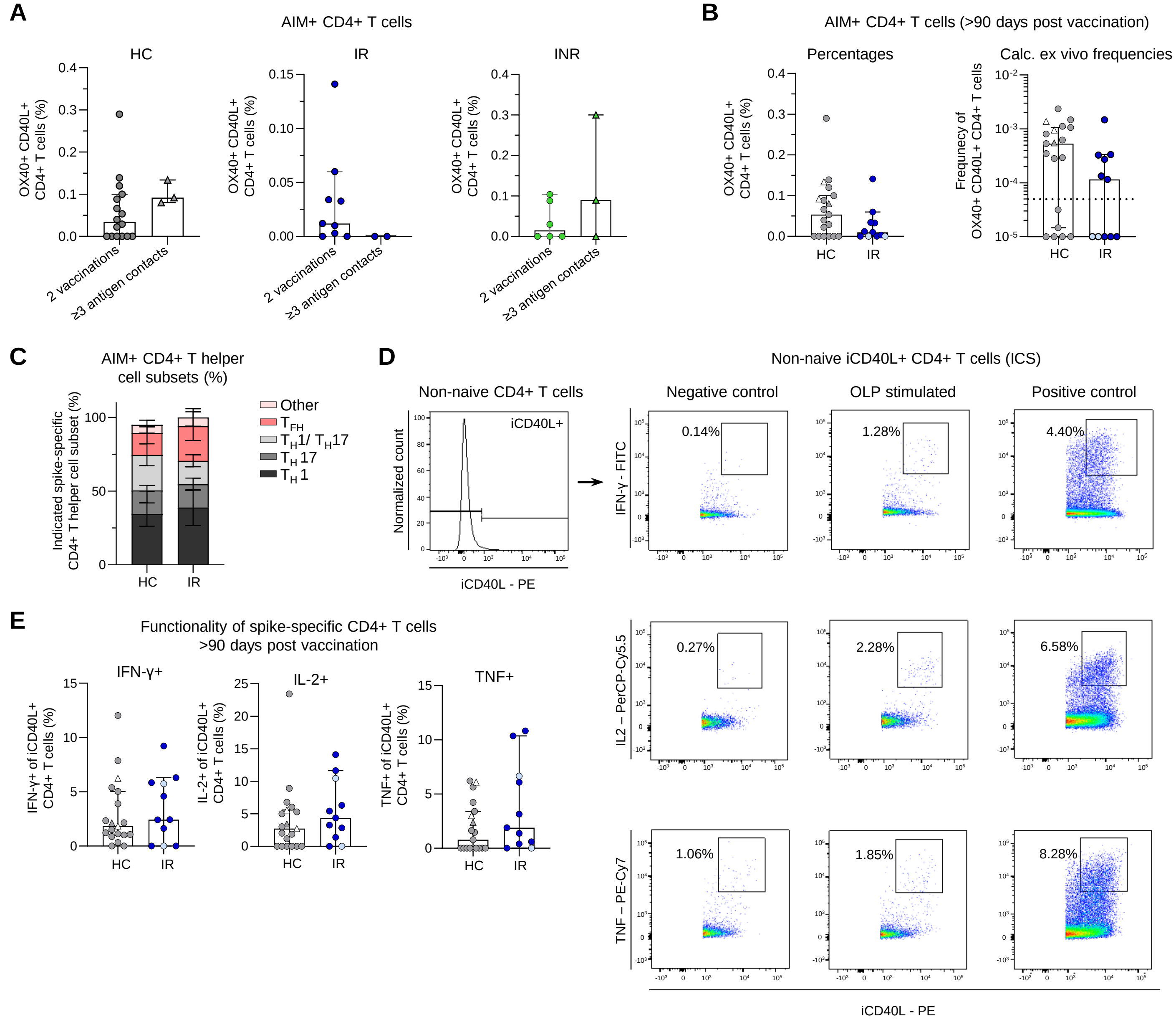

Supplemental Figure 3. Similar virus-specific CD4+ T cell responses in IR and HC. (**A**) Percentages of AIM+ (OX40+ CD40L+) non-naïve CD4+ T cells in HC, IR and INR post 2nd vaccination (HC n=17; IR n=9; INR n=7) and after ≥3 antigen contacts (HC n=2; IR n=2; INR n=2). Values are shown after subtracting the signal detected in paired unstimulated samples. (**B**) Percentages and calculated ex vivo frequencies of AIM+ non-naïve CD4+ T cells in HC (n=19) and IR (n=11) >90 days post 2nd (grey; blue) or 3rd (white; light blue) mRNA vaccination. Values are shown after subtracting the signal detected in paired unstimulated samples. Data from same HC as in main figure 1C are displayed. (**C**) Percentages of non-naive AIM+ CD4+ T helper cell subsets are shown in HC (n=12) and IR (n=2). Data from same HC as in main figure 1D are displayed. (**D**) Gating strategy to detect intracellular cytokine production of spike-reactive non-naive CD4+ T cells, defined by intracellular CD40L (iCD40L) expression. (**E**) Percentages of indicated intracellular cytokine production of iCD40L+ CD4+ T cells after stimulation with a pool of overlapping peptides (OLPs) covering the whole spike protein. Values are shown after subtracting the signal detected in paired unstimulated samples (HC n=19; IR n=11). Data from same HC as in main figure 1E are displayed. Grey and blue indicate time points post 2nd vaccination. White and light blue indicate time points post 3rd vaccination. Median values are depicted with 95% confidence interval error bars. Statistical analysis was performed by a two-tailed Mann–Whitney U test (A, B, E). Circles indicate vaccine-induced CD4+ T cell responses. Triangles indicate hybrid immunity.

Supplemental Figure 4

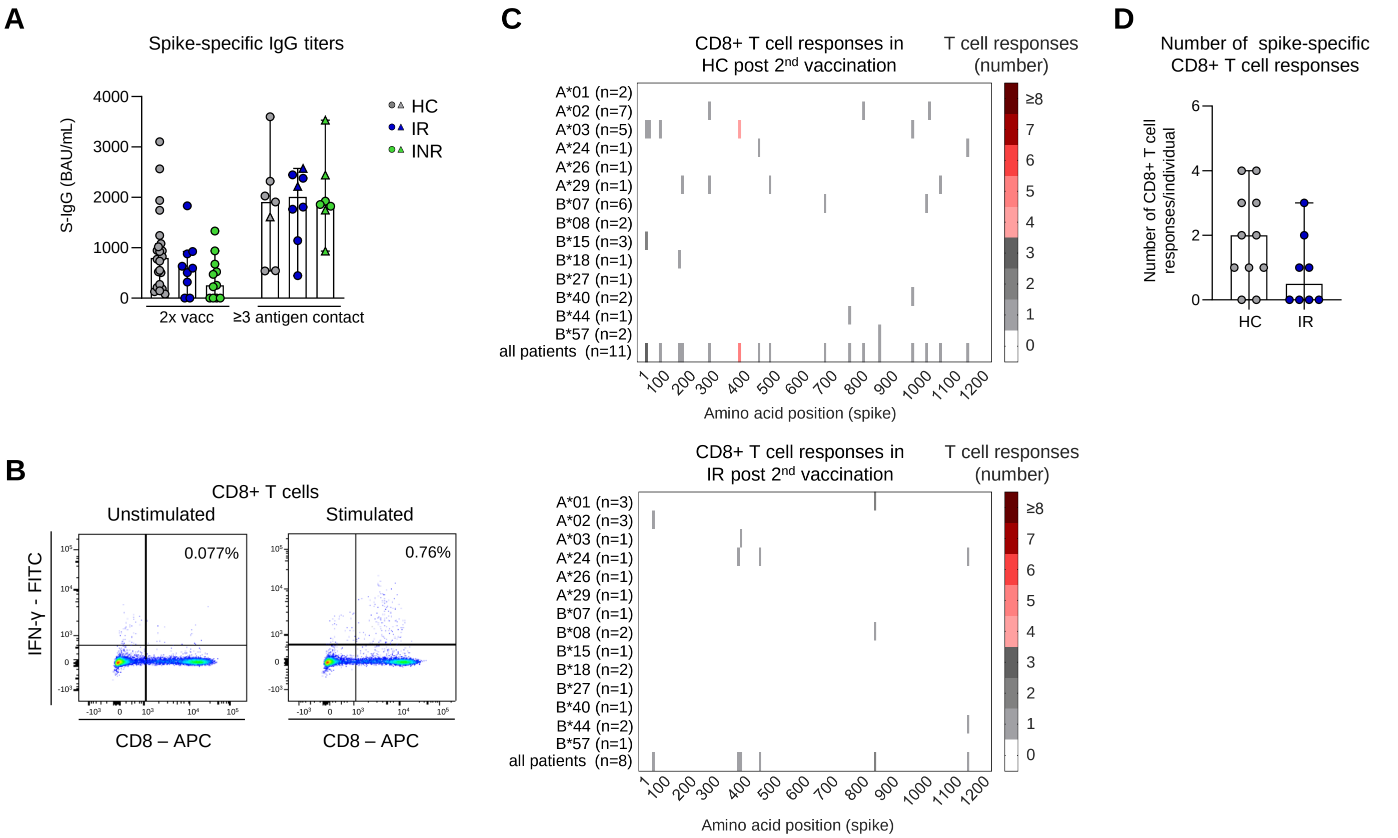

Supplemental Figure 4. Diminished effector response in INR and IR. (**A**) Spike-specific IgG levels at >90 days post 2nd vaccination (HC n=22; IR n=11; INR n=11) and after ≥3 antigen contacts (HC n=7; IR n=8; INR n=7) in HC, IR and INR. ≥3 antigen contacts refer to either 3 vaccinations or a combination of vaccination and infection. (**B**) Representative dot plots of unstimulated and peptide-stimulated CD8+ T cells. (**C**) Heatmap displays number and breadth of spike-specific CD8+ T cell responses to overlapping peptides (OLPs) of the spike protein in HC (n=11) and IR (n=8) >20 days post 2nd vaccination. Number of tested individuals (per HLA allotype and in total) and location of these epitopes within the spike protein are indicated. Data from same HC as in main figure 2A are displayed. (**D**) Number of CD8+ T cell responses per individual induced by stimulation with OLPs. Data from same HC as in main figure 2B are displayed. Median values are depicted with 95% confidence interval error bars. Statistical analysis was performed with a Kruskal-Wallis test and Dunn's multiple comparison test (A) and a two-tailed Mann–Whitney U test (D). Circles indicate vaccine-induced responses. Triangles indicate hybrid immunity.

Supplemental Figure 5

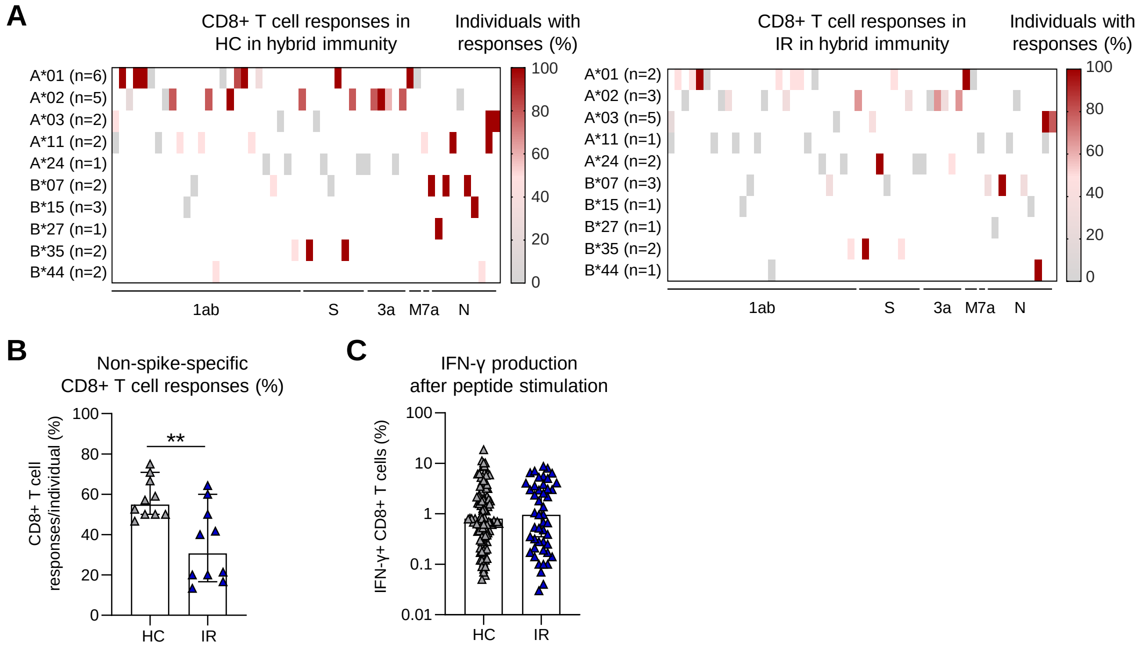

Supplemental Figure 5. Restricted breadth of virus-specific CD8+ T cell responses in IR with hybrid immunity. (**A**) SARS-CoV-2-specific CD8+ T cell responses of HC (n=10) and IR (n=10) after vaccination and infection (hybrid immunity). Percentages of SARS-CoV-2-specific CD8+ T cell responses are shown against epitopes within the complete WT SARS-CoV-2 proteome. These CD8+ T cell epitopes have been described to be restricted by the indicated HLA allotypes. (**B, C**) Percentages of HLA-matched non-spike-specific CD8+ T cell responses per individual (B) and intracellular IFN-γ production (C) after stimulation with pre-described, optimal CD8+ T cell epitopes. Bar graph displays IFN-γ production after peptide-specific stimulation. Values are shown after subtracting the signal detected in paired unstimulated samples. Data from same HC as in main figure 2C-E are displayed, respectively. Median values are depicted with 95% confidence interval error bars. Statistical analysis was performed with a two-tailed Mann–Whitney U test (B, C). Triangles indicate hybrid immunity.

Supplemental Figure 6

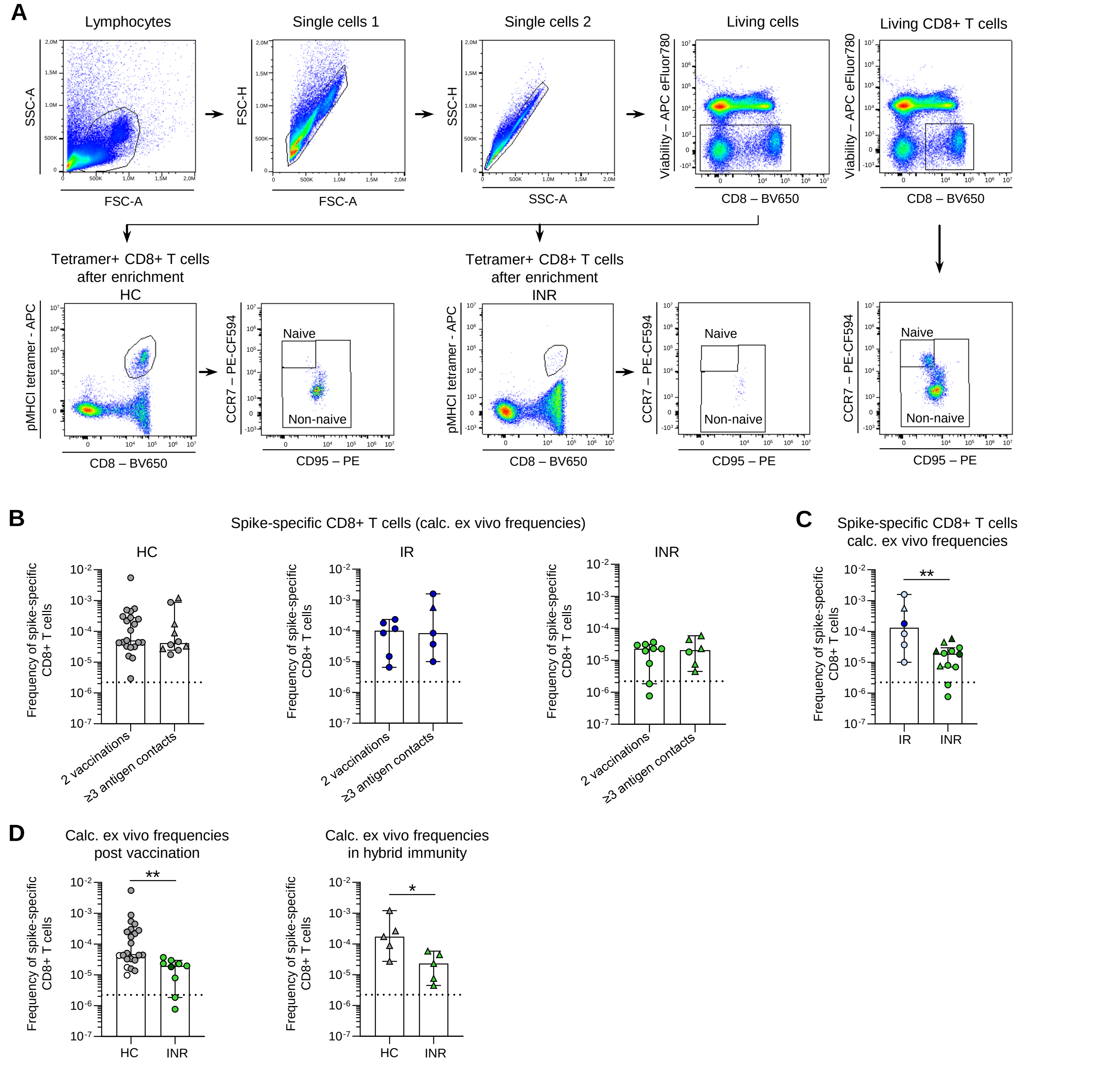

Supplemental Figure 6. Reduced frequencies of virus-specific CD8+ T cells in INR after vaccination and in hybrid immunity. (**A**) Gating strategy to detect SARS-CoV-2 spike-specific CD8+ T cells after pMHC class I tetramer-based enrichment. Exemplary dot plots are shown for spike-specific CD8+ T cells after pMHC class I tetramer-based enrichment in HC and INR. (**B**) Calculated ex vivo frequencies of spike-specific CD8+ T cells post 2nd vaccination and ≥3 antigen contacts in HC, IR and INR (2nd vaccination: HC n=21, IR n=6, INR n=9; ≥3 antigen contacts: HC n=10, IR n=5, INR n=6). (**C**) Calculated ex vivo frequencies of spike-specific CD8+ T cells in IR (n=6) and INR (n=12). Blue and green indicate time points post 2nd vaccination. Light blue and dark green indicate time points post 3rd vaccination. (**D**) Calculated ex vivo frequencies of vaccine-induced (HC n=22; INR n=9) spike-specific CD8+ T cells and after hybrid immunity (HC n=5; INR n=5). Statistical analysis was performed with a two-tailed Mann–Whitney U test (B-D). Circles indicate vaccine-induced CD8+ T cell responses. Triangles indicate hybrid immunity.

Supplemental Figure 7

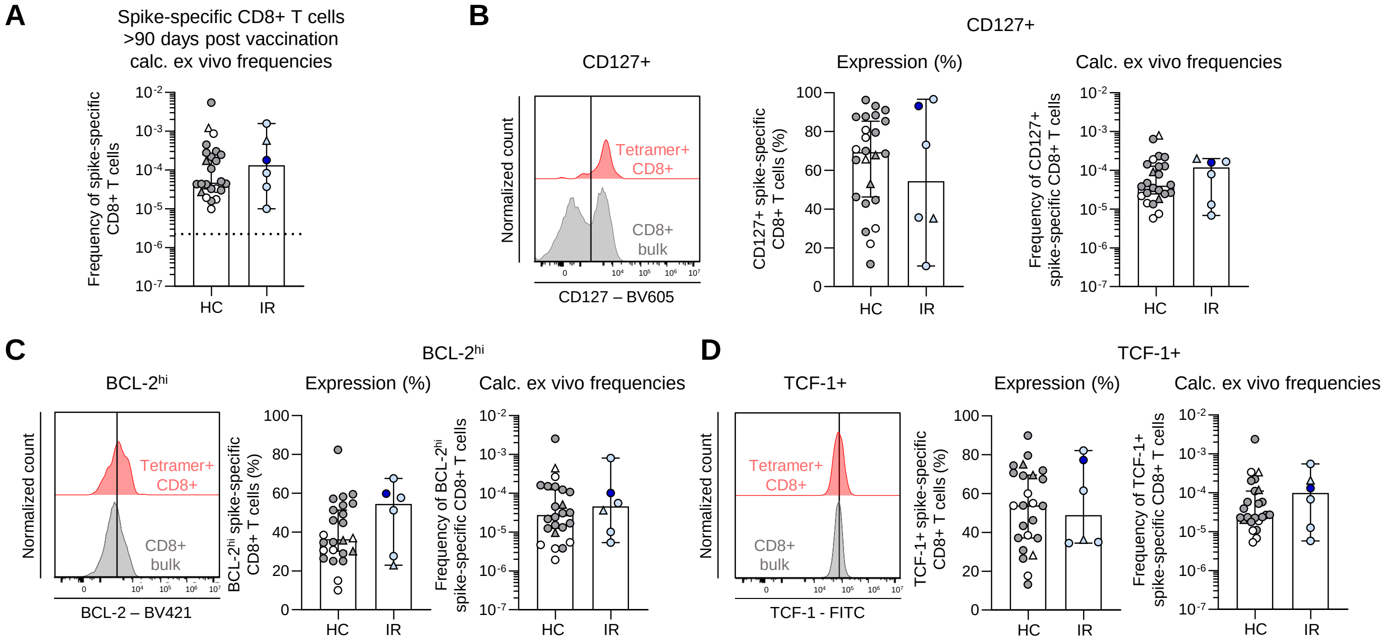

Supplemental Figure 7. Robust memory formation of virus-specific CD8+ T cells in IR. (**A**) Calculated ex vivo frequencies of spike-specific CD8+ T cells in HC (n=24) and IR (n=6). (**B-D**) Percentages and calculated ex vivo frequencies of (B) CD127+, (C) BCL-2^{hi} and (D) TCF-1+ non-naïve spike-specific CD8+ T cells. Grey and blue indicate time points post 2nd vaccination. White and light blue indicate time points post 3rd vaccination. Data from same HC as in main figure 3A-D are displayed, respectively. Median values are depicted with 95% confidence interval error bars. Statistical analysis was performed with a two-tailed Mann–Whitney U test (A-D). Circles indicate vaccine-induced CD8+ T cell responses. Triangles indicate hybrid immunity.

Supplemental Figure 8

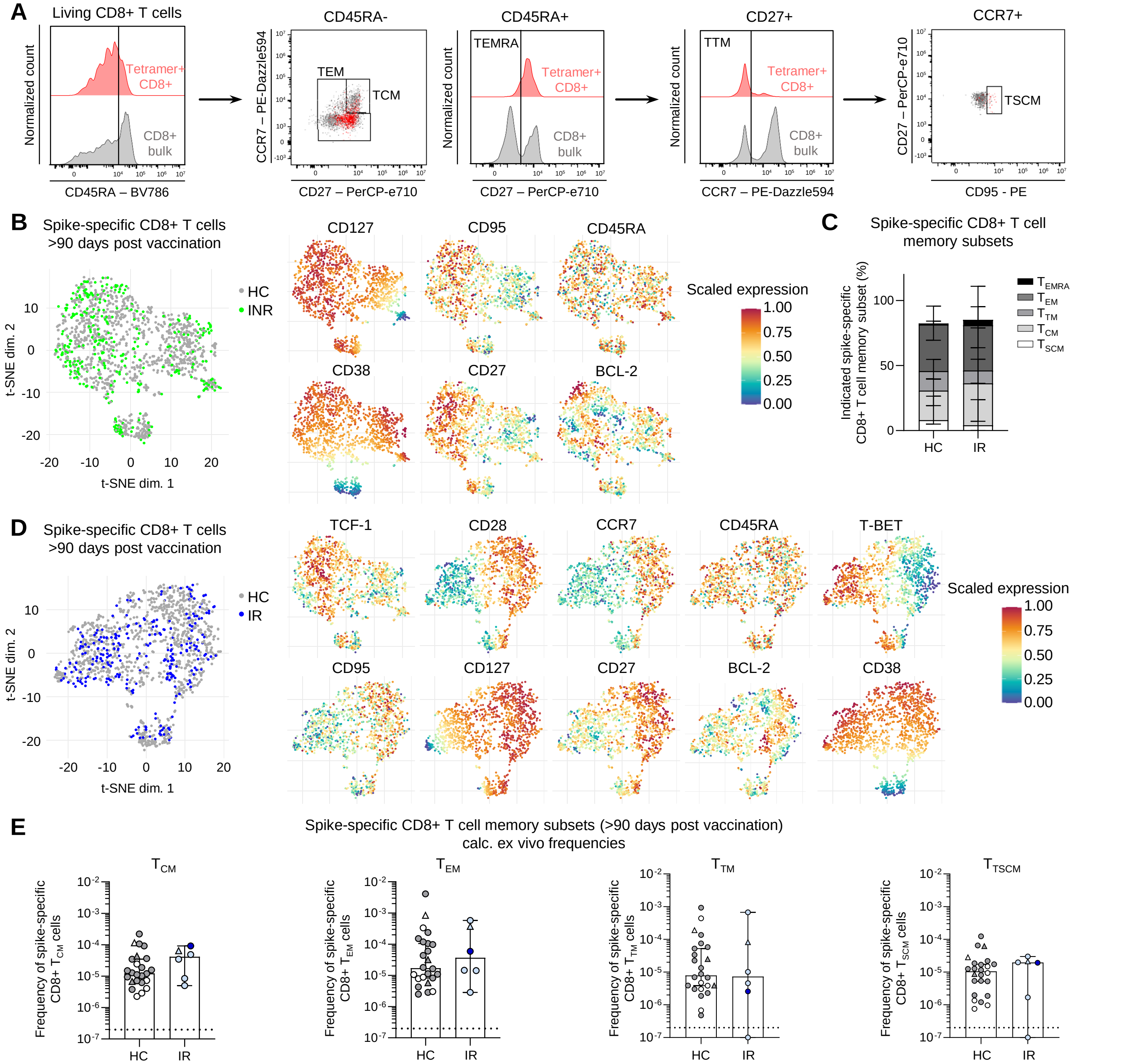

Supplemental Figure 8. Similar subset diversification of virus-specific memory CD8+ T cells in IR and HC. (**A**) Gating strategy to identify indicated CD8+ T cell memory subsets >90 days post vaccination. (**B**) t-SNE representation of flow cytometry data depicting non-naïve spike-specific CD8+ T cells >90 days post 2nd or 3rd mRNA vaccination in HC and INR (grey: HC post 1st boost (n=17) and post 2nd boost (n=6); green: INR post 1st boost (n=7) and post 2nd boost (n=2). (**C**) Percentages of indicated spike-specific CD8+ T cell memory subsets in HC (n=24) and IR (n=6). T_{EMRA}: terminally differentiated effector memory cells re-expressing CD45RA; T_{EM}: effector memory; T_{TM}: transitional memory; T_{CM}: central memory; T_{SCM}: stem cell-like memory. (**D**) t-SNE representation of flow cytometry data depicting non-naïve spike-specific CD8+ T cells >90 days post 2nd or 3rd mRNA vaccination in HC and IR (grey: HC post 1st boost (n=16) and post 2nd boost (n=7); blue: IR post 1st boost (n=1) and post 2nd boost (n=5). (**E**) Calculated ex vivo frequencies of spike-specific CD8+ T cell memory subsets in HC (n=24) and IR (n=6). Grey and blue indicate time points post 2nd vaccination. White and light blue indicate time points post 3rd vaccination. Data from same HC as in main figure 3E-G are displayed (C-E). Median values are depicted with 95% confidence interval error bars. Statistical analysis was performed with a two-tailed Mann–Whitney U test (E). Circles indicate vaccine-induced CD8+ T cell responses. Triangles indicate hybrid immunity.

Supplemental Figure 9

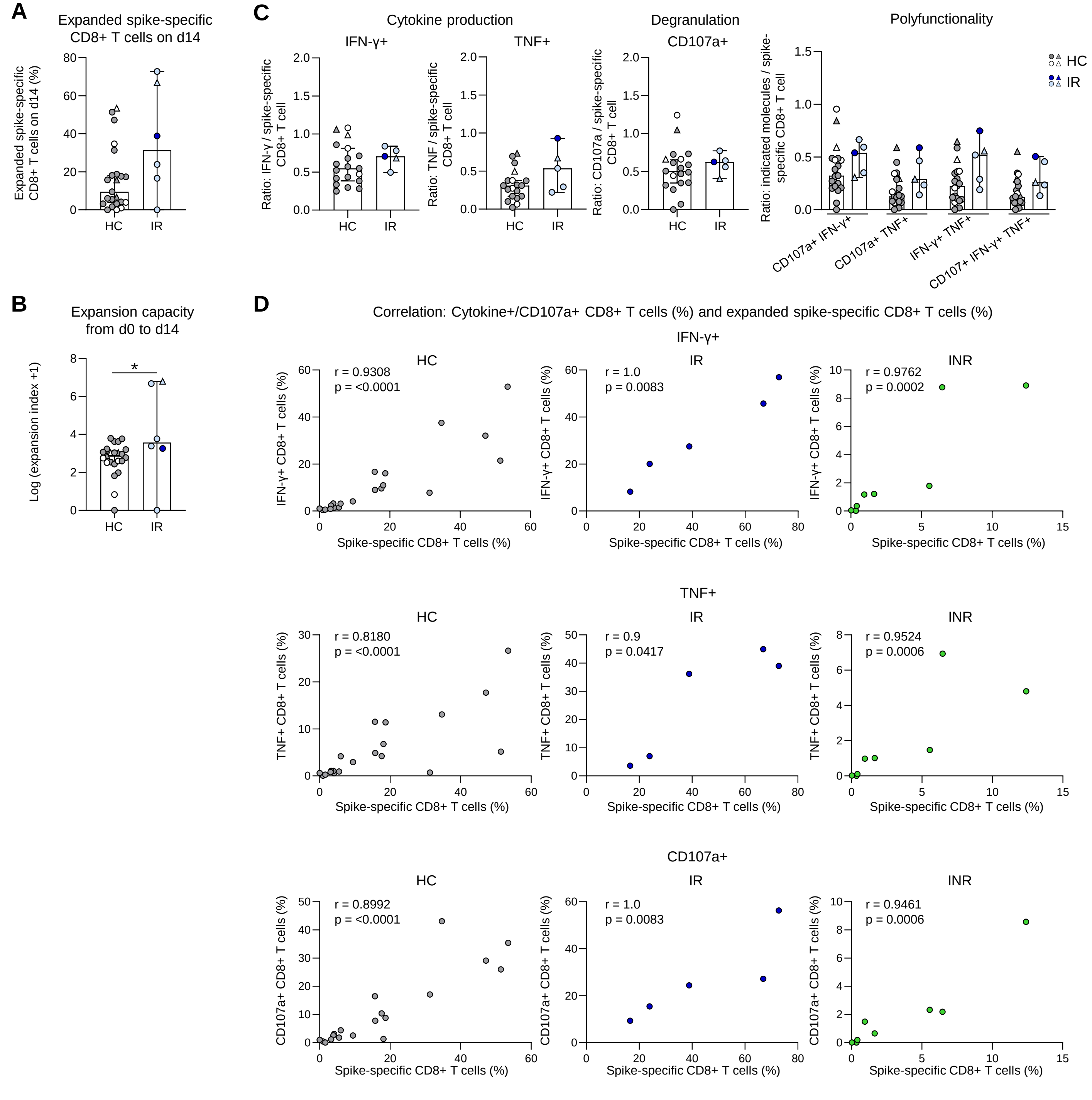

Supplemental Figure 9. Similar in vitro recall responses of virus-specific CD8+ T cells in IR and HC. (**A, B**) Percentages of expanded spike-specific CD8+ T cells (A) and expansion capacity of spike-specific CD8+ T cells (B) after 14 days of in vitro expansion in HC (n=23) and IR (n=6) >90 days post 2nd (grey; blue) or 3rd (white; light blue) mRNA vaccination. (**C**) Percentages of cytokine-producing CD8+ T cells related to the frequency of spike-specific CD8+ T cells after in vitro expansion >90 days post 2nd (grey; blue) or 3rd (white; light blue) mRNA vaccination. (**D**) Correlation of cytokine+/CD107a+ CD8+ T cells (%) and spike-specific CD8+ T cells (%) in HC (n=20), IR (n=5) and INR (n=8). Data from same HC as in main figure 4B-D are displayed (A-C). Median values are depicted with 95% confidence interval error bars. Statistical analysis was performed with a two-tailed Mann–Whitney U test (A-C), a two-way ANOVA with Šídák's multiple comparisons test to compare the polyfunctionality (C) and Spearman correlation (D). Circles indicate vaccine-induced CD8+ T cell responses. Triangles indicate hybrid immunity.

Supplemental Figure 10

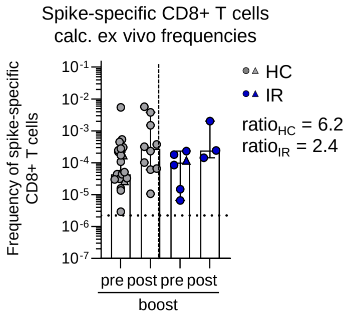

Supplemental Figure 10. Similar in vivo recall responses of virus-specific CD8+ T cells in IR and HC. Calculated ex vivo frequencies of spike-specific CD8+ T cells before (HC n=23; IR n=5) and 6-15 days post (HC n=10; IR n=3) 2nd boost. The ratio is calculated of the median frequency pre versus post 2nd boost of HC and IR, respectively. Data from same HC as in main figure 4E are displayed. Median values are depicted with 95% confidence interval error bars. Statistical analysis was performed with a Kruskal-Wallis test and Dunn's multiple comparison test to compare the frequencies of spike-specific CD8+ T cells pre versus post booster vaccination. Circles indicate vaccine-induced CD8+ T cell responses. Triangles indicate hybrid immunity.

Supplemental Figure 11

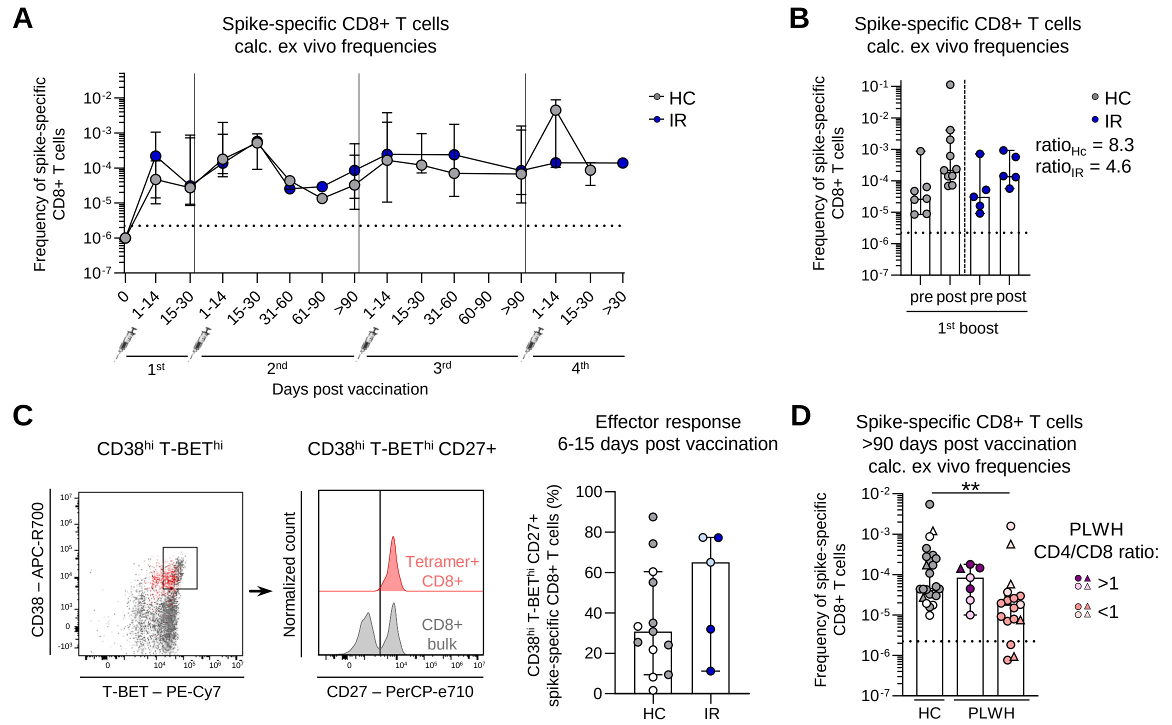

Supplemental Figure 11. Similar activation and dynamics of spike-specific CD8+ T cell responses in IR and HC. (**A**) Calculated ex vivo frequencies of spike-specific CD8+ T cells throughout 1st, 2nd, 3rd and 4th mRNA vaccination. Median is depicted of HC (n=9 post 3rd vaccination, of which n=3 are post 4th vaccination) and IR (n=5 post 3rd vaccination, of which n=2 are post 4th vaccination) (**B**) Calculated ex vivo frequencies of spike-specific CD8+ T cells before (HC n=7; IR n=5) and 6-15 days post (HC n=10; IR n=5) 1st boost. The ratio is calculated of the median frequency pre versus post boost of HC and IR, respectively. (**C**) Representative plots and percentages are shown of CD38hi T-BEThi CD27+ non-naïve spike-specific CD8+ T cells 6-15 days post booster vaccination in HC (n=13) and IR (n=5). Grey and blue indicate time points post 2nd vaccination. White and light blue indicate time points post 3rd vaccination. (**D**) Calculated ex vivo frequencies of spike-specific CD8+ T cells are depicted from HC (n=24) and PLWH (n=24) >90 days post 2nd (dark colors) or 3rd (light colors) mRNA vaccination. Frequencies of spike-specific CD8+ T cells of PLWH are shown depending on their CD4/CD8 ratio (>1 n= 7; <1 n=17). Data from same HC as in main figure 6A-C (A-C) and 6G (D) are displayed. Median values are depicted with 95% confidence interval error bars. Statistical analysis was performed with a Kruskal-Wallis test and Dunn's multiple comparison test (B, D) and a two-tailed Mann–Whitney U test (C). Circles indicate vaccine-induced CD8+ T cell responses. Triangles indicate hybrid immunity.

Supplemental Table 1: Characteristics of HIV-infected immunological non-responders (INR)

Donor ID	Sex	Age	HLA type	CD4+ T cell count	CDC Stadium	CD4/CD8 ratio	HIV RNA
P1	m	48	A*0301, A*2402, B*2705, B*4901, DQB1*0501, DQB1*0604, DRB1*0101, DRB1*1302	194	B3	0.39	<20
P2	m	56	A*2402, B*1302, B*2705, DQB1*0202, DQB1*0503, DRB1*07101, DRB1*1454	300	C3	1.16	<20
P3	m	32	A*0201, A*0301, B*0702, B*3801, DQB1*0301, DQB1*0603, DRB1*1101, DRB1*1301	152	B3	0.18	<20
P4	m	54	A*0101, A*0201, B*1501, DQB1*0501, DQB1*0603, DRB1*0101, DRB1*1301	129	C3	0.33	<20
P5	f	57	A*0101, A*0201, B*0801, B*3701, DQB1*0201, DQB1*0502, DRB1*0301, DRB1*1501	298	C3	0.31	<20
P6	m	35	A*2301, A*3001, B*4202, B*5301, DQB1*0301, DQB1*0319, DRB1*0804, DRB1*1304	89	B3	0.04	6010
P7	f	43	A*0101, B*0801, DQB1*0201, DRB1*0301	153		0.2	<20
P8	m	39	A*1101, A*2402, B*4405, B*5501, DQB1*0501, DQB1*0503, DRB1*0101, DRB1*1454	287	A3	0.3	<20
P9	m	43	A*0201, A*2301, B*0706, B*5101, DQB1*0202, DQB1*0301, DRB1*0901, DRB1*1303	277	B2	1.25	<20
P10	m	52	A*0301, B*1801, B*4403, DQB1*0202, DQB1*0301, DRB1*0701, DRB1*1201	287	C3	0.3	<20
P11	m	34	A*0201, A*0202, B*4501, B*5102, DQB1*0202, DQB1*0319, DRB1*0901, DRB1*1102	114	A3	0.14	<20
P12	f	52	A*0301, A*2301, B*0702, B*1503, DQB1*0319, DQB1*0602, DRB1*1101, DRB1*1501	202		0.22	<20
P13	m	73	A*1101, A*2402, B*1513, B*3802, DQB1*0301, DRB1*1202	186	C3	0.35	<20
P14	m	61	A*0101, A*6801, B*0702, B*0801, DQB1*0201, DQB1*0602, DRB1*0301, DRB1*1501	295	B2	1.33	<20
P15	m	53	A*0201, B*3501, B*4405, DQB1*0302, DQB1*0402, DRB1*0404, DRB1*0801	157	C3	0.27	<20
P16	m	28	A*3303, A*8001, B*0801, B*5801, DQB1*0501, DQB1*0609, DRB1*1001, DRB1*1302	62	A3	0.12	73
P17	m	38	A*0201, A*2902, B*4402, B*4403, DQB1*0202, DQB1*0301, DRB1*0401, DRB1*0701	144	C3	0.17	<20

Supplemental Table 2: Characteristics of HIV-infected immunological responders (IR)

Donor ID	Sex	Age	HLA type	CD4+ T cell count	CDC Stadium	CD4/CD8 ratio	HIV RNA
P18	m	49	A*0201, A*0301, B*4402, B*5601, DQB1*0602, DQB1*0603, DRB1*1301, DRB1*1501	506	C3	0.59	<20
P19	m	41	A*0101, A*1101, B*1803, B*5001, DQB1*0202, DQB1*0501, DRB1*0101, DRB1*0701	1262	B1	1.68	<20
P20	m	53	A*0301, A*2902, B*4901, B*8101, DQB1*0301, DQB1*0302, DRB1*0405, DRB1*1303	708	C3	0.62	<20
P21	m	46	A*0201, B*1501, B*1801, DQB1*0301, DQB1*0501, DRB1*0101, DRB1*1104	846	B1	1.13	<20
P22	m	35	A*1101, A*2902, B*0702, B*2705, DQB1*0501, DQB1*0602, DRB1*0101, DRB1*1501	994	B1	0.82	<20
P23	m	58	A*0201, B*0702, B*1801, DQB1*0301, DQB1*0604, DRB1*1104, DRB1*1301	640	A3	0.75	<20
P24	m	61	A*2301, A*7401, B*0702, B*5001, DQB1*0202, DQB1*0602, DRB1*0701, DRB1*1503	563	B2	0.74	<20
P25	m	47	A*0201, A*0301, B*4403, B*4901, DQB1*0301, DQB1*0501, DRB1*0102, DRB1*0803	716	A2	1.49	<20
P26	f	39	A*0301, A*2601, B*07199, B*3801, DQB1*0302, DQB1*0602, DRB1*0404, DRB1*1501	450	C3	1.17	<20
P27	m	36	A*2402, A*7406, B*40, B*44, DQB1*0501, DQB1*0602, DRB1*0101, DRB1*1501	518	C3	0.65	<20
P28	m	63	A*0301, A*3201, B*1517, B*2705, DQB1*0301, DQB1*0604, DRB1*0401, DRB1*1302	611	A3	0.91	<20
P29	m	89	A*0301, A*1101, B*4427, B*5101, DQB1*0402, DQB1*0502, DRB1*0801, DRB1*1601	743	B2	2.38	<20
P30	f	40	A*0101, A*0301, B*0801, B*3501, DQB1*0201, DQB1*0501, DRB1*0101, DRB1*0301	513	A2	1.27	<20
P31	m	56	A*0201, B*0702, B*1302, DQB1*0501, DQB1*0503, DRB1*0101, DRB1*1454	1018	A2	1.51	<20
P32	m	28	A*0201, A*0202, B*0801, B*3501, DQB1*0319, DRB1*1101, DRB1*1304	670	C2	2.74	<20
P33	m	28	A*0201, A*0301, B*0702, B*3501, DQB1*0301, DQB1*0602, DRB1*1101, DRB1*1501	468		1.27	<20
P34	m	49	A*0101, A*0201, B*2702, B*4405, DQB1*0501, DQB1*0502, DRB1*0101, DRB1*1601	931	A2	2.39	<20
P35	m	44	A*0201, A*3201, B*1801, B*5107, DQB1*0301, DQB1*0502, DRB1*1101, DRB1*1602	426	A2	0.49	<20
P36	m	54	A*0101, A*0201, B*0801, B*3701, DQB1*0302, DQB1*0303, DRB1*0401, DRB1*0701	575	A2	0.61	<20
P37	m	58	A*0301, A*2402, B*0702, B*4405, DQB1*0501, DQB1*0503, DRB1*0101, DRB1*1454	587	A2	0.81	<20
P38	m	48	A*0201, A*3402, B*4402, B*4403, DQB1*0602, DRB1*1501, DRB1*1503	1106	B2	1.67	<20
P39	m	51	A*1101, A*2601, B*3501, B*5701, DQB1*0303, DQB1*0501, DRB1*0101, DRB1*0701	976	A1	1.33	<20
P40	m	43	A*0101, A*0301, B*0801, B*3503, DQB1*0201, DQB1*0503, DRB1*0301, DRB1*1454	777	A2	0.89	<20
P41	m	79	A*0101, A*2402, B*0801, B*2705, DQB1*0201, DQB1*0503, DRB1*0301, DRB1*1454	354		0.37	<20
P42	m	41	A*2501, A*3201, B*3503, B*5101, DQB1*0302, DQB1*0402, DRB1*0401, DRB1*0801	842	A2	0.86	<20
P43	f	62	A*0101, A*0201, B*0702, B*5701, DQB1*0303, DRB1*0701	407	B3	0.72	<20
P44	f	62	A*0101, A*0201, B*4402, B*5701, DQB1*0302, DQB1*0602, DRB1*0401, DRB1*1501	328	C3	0.29	<20

Supplemental Table 3: Overview of SARS-CoV-2 infection history in people living with HIV and immunological analyses

Donor ID	Cohort	Infection prior vaccination	Infection post vaccination	CD8+ T cell analyses	CD4+ T cell analyses	Serum S-IgG1 assay
P1	INR		07/2022	yes	yes	yes
P2	INR		yes	yes	yes	yes
P3	INR		10/2022; 03/2023	yes		yes
P4	INR		07/2022	yes		yes
P5	INR		spring 2022	yes	yes	yes
P6	INR		yes	yes	yes	
P7	INR			yes	yes	yes
P8	INR		07/2022	yes	yes	
P9	INR	yes	yes	yes	yes	yes
P10	INR		02/2022	yes	yes	yes
P11	INR	yes		yes		yes
P12	INR		07/2022	yes	yes	yes
P13	INR			yes	yes	
P14	INR		09/2022	yes	yes	yes
P15	INR			yes	yes	yes
P16	INR			yes	yes	yes
P17	INR			yes	yes	yes
P18	IR			yes	yes	yes
P19	IR			yes	yes	yes
P20	IR			yes	yes	yes
P21	IR			yes	yes	yes
P22	IR			yes	yes	
P23	IR			yes	yes	yes
P24	IR		yes	yes		
P25	IR		03/2022	yes	yes	yes
P26	IR			yes		
P27	IR		10/2022	yes	yes	
P28	IR		winter 2021/2022	yes		
P29	IR		03/2022	yes		
P30	IR		07/2022	yes	yes	yes
P31	IR		04/2023	yes	yes	yes
P32	IR		10/2021	yes		
P33	IR		01/2022	yes		
P34	IR			yes	yes	yes
P35	IR			yes		
P36	IR			yes	yes	yes
P37	IR		03/2022	yes		
P38	IR			yes	yes	
P39	IR			yes	yes	
P40	IR		12/2021	yes	yes	
P41	IR	yes	10/2022	yes		
P42	IR			yes	yes	yes
P43	IR			yes		
P44	IR			yes		

Supplemental Table 4: Characteristics of HIV-uninfected, healthy controls (HC)

Donor ID	Sex	Age	HLA type
H1	m	41	A*0101, A*0301, B*0801, B*3501, DQB1*0201, DQB1*0501, DRB1*0101, DRB1*0301
H2	m	31	A*0201, A*0301, B*1501, B*5703, DQB1*0302, DQB1*0602, DRB1*0401, DRB1*1401
H3	f	38	A*0201, A*2601, B*0702, B*3801, DQB1*0602, DQB1*0603, DRB1*1301, DRB1*1501
H4	f	30	A*2601, B*0702, B*3801, DQB1*0302, DQB1*0602, DRB1*0401, DRB1*1501
H5	m	43	A*0301, A*3201, B*0702, B*4002, DQB1*0301, DQB1*0503, DRB1*1101, DRB1*1454
H6	m	69	A*0201, B*1302, B*1501, DQB1*0202, DQB1*0302, DRB1*0401, DRB1*0701
H7	f	48	A*0301, A*6901, B*3508, B*5101, DQB1*0502, DQB1*0603, DRB1*1301, DRB1*1602
H8	m	41	A*0101, A*0201, B*0801, B*4001, DQB1*0301, DQB1*0602, DRB1*1402, DRB1*1501
H9	f	35	A*0101, A*0301, B*0702, B*5701, DQB1*0303, DQB1*0602, DRB1*0701, DRB1*1501
H10	f	27	A*0201, B*0702, B*0801, DQB1*0201, DQB1*0402, DRB1*0301, DRB1*0801
H11	m	48	A*0101, A*1101, B*0801, B*1501, DQB1*0201, DQB1*0501, DRB1*0101, DRB1*0301
H12	m	32	A*0301, B*0702, B*4402, DQB1*0301, DQB1*0603, DRB1*11198, DRB1*1501
H13	f	60	A*0201, B*0702, B*4402, DQB1*0301, DQB1*0602, DRB1*0401, DRB1*1501
H14	f	29	A*0101, A*3301, B*1402, B*5701, DQB1*0201, DQB1*0602, DRB1*0301, DRB1*1501
H15	f	44	A*0101, A*1101, B*1517, B*3501, DQB1*0501, DQB1*0604, DRB1*0103, DRB1*1302
H16	m	31	A*0101, A*0301, B*0801, B*1801, DQB1*0201, DQB1*0603, DRB1*0301, DRB1*1301
H17	m	47	A*0201, A*2402, B*2705, B*5101, DQB1*0301, DRB1*1101, DRB1*1104
H18	f	50	A*0301, B*0702, B*2705, DQB1*0301, DQB1*0603, DRB1*1303, DRB1*1501
H19	f	28	A*0101, A*0301, B*2705, B*3701, DQB1*0301, DQB1*0501, DRB1*1001, DRB1*1201
H20	f	48	A*0101, A*2402, B*1302, B*5701, DQB1*0202, DQB1*0301, DRB1*0701, DRB1*1201
H21	f	26	A*0201, A*3101, B*0702, B*4501, DQB1*0301, DQB1*0602, DRB1*0401, DRB1*1501
H22	m	60	A*0201, B*0801, B*1501, DQB1*0201, DQB1*0301, DRB1*0301, DRB1*1101
H23	m	63	A*0101, A*0201, B*0801, B*1501, DQB1*0301, DQB1*0402, DRB1*0801, DRB1*1101
H24	f	52	A*0201, A*6801, B*1501, B*4402, DQB1*0301, DQB1*0503, DRB1*1103, DRB1*1454
H25	f	52	A*0101, A*2402, B*0801, B*2705, DQB1*0202, DQB1*0501, DRB1*0101, DRB1*0701
H26	m	35	A*0201, A*6801, B*1501, B*5101, DQB1*0302, DQB1*0501, DRB1*0101, DRB1*0401
H27	m	25	A*0201, A*2902, B*4501, B*5101, DQB1*0301, DQB1*0603, DRB1*0401, DRB1*1301
H28	f	32	A*0101, B*5701, DQB1*0301, DQB1*0303, DRB1*0701, DRB1*1101
H29	m	31	A*0301, A*3001, B*1302, B*3501, DQB1*0301, DQB1*0602, DRB1*1201, DRB1*1501
H30	m	36	A*0101, A*2402, B*0702, B*2705, DQB1*0502, DQB1*0602, DRB1*1501, DRB1*1601
H31	f	78	A*0201, A*0301, B*0702, B*1801, DQB1*0503, DQB1*0602, DRB1*1454, DRB1*1501
H32	m	25	A*0301, A*6801, B*1402, B*4402, DQB1*0301, DQB1*0609, DRB1*1201, DRB1*1302
H33	f	26	A*0301, A*7401, B*0702, B*1503, DQB1*0301, DQB1*0602, DRB1*1101, DRB1*1501
H34	f	24	A*0101, A*0301, B*0801, B*3501, DQB1*0302, DQB1*0501, DRB1*0101, DRB1*0401
H35	f	55	A*0101, A*0201, B*0801, B*1501, DQB1*0302, DQB1*0303, DRB1*0401, DRB1*0901
H36	m	27	A*0101, A*2501, B*0801, B*4402, DQB1*0501, DRB1*0101
H37	f	22	A*0101, A*0201, B*1501, B*4403, DQB1*0202, DQB1*0603, DRB1*0701, DRB1*1301
H38	f	27	A*0201, A*0202, B*4101, B*5701, DQB1*0603, DQB1*0609, DRB1*1301, DRB1*1302
H39	f	21	A*0201, A*1101, B*3501, B*5101, DQB1*0402, DQB1*0501, DRB1*0101, DRB1*0801

Supplemental Table 5: Overview of SARS-CoV-2 infection history in heathy controls and immunological analyses

Donor ID	Cohort	Infection post vaccination	CD8+ T cell analyses	CD4+ T cell analyses	Serum S-IgG1 assay
H1	HC	01/2022	yes	yes	
H2	HC		yes	yes	yes
H3	HC	03/2022	yes	yes	yes
H4	HC		yes	yes	
H5	HC		yes	yes	
H6	HC		yes	yes	yes
H7	HC		yes	yes	yes
H8	HC		yes	yes	yes
H9	HC		yes	yes	
H10	HC		yes	yes	yes
H11	HC	02/2022	yes	yes	yes
H12	HC		yes	yes	yes
H13	HC		yes	yes	yes
H14	HC	05/2022	yes	yes	yes
H15	HC		yes		yes
H16	HC		yes	yes	yes
H17	HC		yes	yes	
H18	HC		yes	yes	yes
H19	HC		yes	yes	yes
H20	HC		yes		
H21	HC		yes	yes	yes
H22	HC		yes	yes	yes
H23	HC	11/2021	yes	yes	yes
H24	HC		yes	yes	yes
H25	HC	yes	yes		yes
H26	HC		yes		yes
H27	HC		yes	yes	
H28	HC	yes	yes		yes
H29	HC		yes		yes
H30	HC	01/2022	yes		
H31	HC		yes	yes	
H32	HC	03/2022	yes	yes	yes
H33	HC	yes	yes	yes	yes
H34	HC		yes	yes	yes
H35	HC		yes		yes
H36	HC	yes	yes		
H37	HC	01/2022	yes		
H38	HC	04/2022	yes		
H39	HC	05/2022	yes		

Supplemental Table 6: In this study analyzed optimal CD8+ T cell epitopes

Epitope	Amino acid sequence
A*03/11/ORF1ab$_{808-816}$	VTNNTFTLK
A*01/ORF1ab$_{1321-1329}$	PTDNYITTY
A*02/ORF1ab$_{1566-1574}$	RTIKVFTTV
A*01/ORF1ab$_{1636-1646}$	HTTDPSFLGRY
A*01/ORF1ab$_{1637-1646}$	TTDPSFLGRY
A*01/ORF1ab$_{1889-1899}$	CTEIDPKLDNY
A*11/ORF1ab$_{2192-2200}$	ASMPTTIAK
A*02/ORF1ab$_{2297-2307}$	SLDTYPSLETI
A*02/ORF1ab$_{2332-2340}$	ILFTRFFYV
A*11/ORF1ab$_{2600-2608}$	STFNVPMEK
B*15/ORF1ab$_{2788-2796}$	YLITPVHVM
B*07/ORF1ab$_{2949-2956}$	RPDTRYVL
A*11/ORF1ab$_{3622-3630}$	SAFAMMFVK
A*02/ORF1ab$_{3886-3894}$	KLWAQCVQL
B*44/ORF1ab$_{3946-3954}$	SEFSSLPSY
A*01/ORF1ab$_{4082-4091}$	NTCDGTTFTY
A*02/ORF1ab$_{4094-4102}$	ALWEIQQVV
A*01/ORF1ab$_{4163-4171}$	CTDDNALAY
A*01/ORF1ab$_{4163-4172}$	CTDDNALAYY
A*11/ORF1ab$_{4216-4224}$	VTDTPKGPK
A*01/ORF1ab$_{5130-5138}$	DTDFVNEFY
A*24/ORF1ab$_{5137-5145}$	FYAYLRKHF
B*07/ORF1ab$_{5196-5924}$	IPRRNVATL
A*03/ORF1ab$_{5533-5542}$	VVYRGTTTYK
A*24/ORF1ab$_{5721-5729}$	VYIGDPAQL
B*35/S$_{84-92}$	LPFNDGVYF
A*02/S$_{269-277}$	YLQPRTFLL
B*35/S$_{321-329}$	QPTESIVRF
A*03/S$_{378-386}$	KCYGVSPTK
A*24/S$_{448-456}$	NYNYLYRLF
B*07/S$_{680-688}$	SPRRARSVA
A*01/S$_{865-873}$	LTDEMIAQY
B*35/S$_{896-904}$	IPFAMQMAY
A*02/S$_{1000-1008}$	RLQSLQTYV
A*24/S$_{1208-1216}$	QYIKWPWYI
A*24/S$_{1211-1220}$	KWPWYIWLGF
A*02/ORF3a$_{72-80}$	ALSKGVHFV
A*02/ORF3a$_{82-90}$	NLLLLFVTV
A*02/ORF3a$_{107-115}$	YLYALVYFL
A*24/ORF3a$_{112-120}$	VYFLQSINF
A*02/ORF3a$_{139-147}$	LLYDANYFL
A*01/ORF3a$_{207-215}$	FTSDYYQLY
A*01/M$_{171-179}$	ATSRTLSYY
A*11/M$_{171-180}$	ATSRTLSYYK

Epitope	Amino acid sequence
B*07/ORF7a$_{78-86}$	RARSVSPKL
B*27/N$_{9-17}$	QRNAPRITF
B*07/N$_{105-113}$	SPRWYFYYL
A*11/N$_{134-143}$	ATEGALNTPK
A*02/N$_{222-230}$	LLLDRLNQL
B*07/N$_{257-265}$	KPRQKRTAT
B*15/N$_{305-314}$	AQFAPSASAF
B*44/N$_{322-330}$	MEVTPSGTW
A*03/11/N$_{361-369}$	KTFPPTEPK
A*03/N$_{361-370}$	KTFPPTEPKK

Supplemental Table 7: List of antibodies

Antigen	Conjugate	Clone	Dilution	Isotype	Catalogue number	Manufacturer
CCR7	PE/Dazzle594	G043H7	1:50	Mouse IgG2a, κ	353236	BioLegend
CCR7	BV785	G043H7	1:25	Mouse IgG2a, k	353230	BioLegend
CD8	BV650	RPA-T8	1:200	Mouse IgG1, k	301042	BioLegend
CD8	BV510	SK1	1:100	Mouse BALB/c IgG1, κ	563919	BD Biosciences
CD8	BV421	RPA-T8	1:200	Mouse IgG1, k	562428	BD Biosciences
CD8	APC	SK1	1:300	Mouse BALB/c IgG1, κ	345775	BD Biosciences
CD28	BV510	CD28.2	1:33	Mouse IgG1, k	302936	BioLegend
CD127	BV605	A019D5	1:100	Mouse IgG1, k	351334	BioLegend
CD38	APC-R700	HIT2	1:400	Mouse IgG1, κ	564979	BD Biosciences
CD45RA	BV785	HI100	1:400	Mouse IgG2b, k	304139	BioLegend
CD45RA	BUV496	HI100	1:800	Mouse IgG2b, κ	750258	BD Biosciences
CD95	PE	DX2	1:16.67	Mouse IgG1, k	340480	BD Biosciences
BCL-2	BV421	100	1:200	Mouse IgG1, k	658709	BioLegend
TCF1	AlexaFluor488	C63D9	1:100	Rabbit IgG	6444	Cell Signaling
T-BET	PE-Cy7	4B10	1:200	Mouse IgG1, k	25-5825	eBioscience
CD27	erCP-eFluor710	O323	1:100	Mouse IgG1, k	46-0279-42	eBioscience
CD27	BV605	L128	1:50	Mouse BALB/c IgG1, κ	562655	BD Biosciences
CD4	BUV395	SK3	1:100	Mouse IgG1, k	563552	BD Biosciences
CD4	eFluor450	RPA-T4	1:250	Mouse IgG1, k	48-0049	eBioscience
CCR6	BUV737	11A9	1:25	Mouse IgG1, k	564377	BD Biosciences
CXCR5	BV421	J252D4	1:100	Mouse IgG1, k	356920	BioLegend
Ox40	BV510	L106	1:200	Mouse BALB/c IgG1, κ	745040	BD Biosciences
CD154 (CD40L)	PE	TRAP1	1:20	Mouse BALB/c IgG1, κ	555700	BD Biosciences
CXCR3	APC	G043H7	1:100	Mouse IgG1, k	353712	BioLegend
IFN-γ	FITC	25723.11	1:8	Mouse IgG2b	340449	BD Biosciences
IL-2	PerCP-Cy5.5	MQ1-17H12	1:50	Rat IgG2a, k	500322	BioLegend
TNF	PE-Cy7	MAb11	1:50	Mouse IgG1, κ	557647	BD Biosciences
CD107a	APC	H4A3	1:100	Mouse IgG1, k	560664	BD Biosciences
CD14	APC-eFluor780	61D3	1:400	Mouse IgG1, k	47-0149-42	eBioscience
CD19	APC-eFluor780	HIB19	1:400	Mouse IgG1, k	47-0199-42	eBioscience
viability dye	eFluor780		1:400		65-0865	eBioscience
viability dye	eFluor506		1:300		65-0866	eBioscience
